# Marine self-potential survey for exploring seafloor hydrothermal ore deposits

**DOI:** 10.1038/s41598-017-13920-0

**Published:** 2017-10-19

**Authors:** Yoshifumi Kawada, Takafumi Kasaya

**Affiliations:** 1 Project Team for Development of New-generation Research Protocol for Submarine Resources, Japan Agency for Marine-Earth Science and Technology, 2-15 Natsushima-cho, Yokosuka, 237-0061 Japan; 2 International Research Institute of Disaster Science, Tohoku University, 468-1 Aoba, Aoba-ku Sendai, 980-0845 Japan; 3 Research and Development Center for Earthquake and Tsunami, Japan Agency for Marine–Earth Science and Technology, 2-15 Natsushima-cho, Yokosuka, 237-0061 Japan

## Abstract

We conducted a self-potential survey at an active hydrothermal field, the Izena hole in the mid-Okinawa Trough, southern Japan. This field is known to contain Kuroko-type massive sulphide deposits. This survey measured the self-potential continuously in ambient seawater using a deep-tow array, which comprises an electrode array with a 30-m-long elastic rod and a stand-alone data acquisition unit. We observed negative self-potential signals not only above active hydrothermal vents and visible sulphide mounds but also above the flat seafloor without such structures. Some signals were detectable >50 m above the seafloor. Analysis of the acquired data revealed these signals’ source as below the seafloor, which suggests that the self-potential method can detect hydrothermal ore deposits effectively. The self-potential survey, an easily performed method for initial surveys, can identify individual sulphide deposits from a vast hydrothermal area.

## Introduction

Seafloor hydrothermal systems often have accompanying sulphide ore deposits^1^, which have been regarded as submarine mineral resources to be explored and mined^2^. The targets for mining include ore deposits associated with both active and inactive hydrothermal systems. Ore deposits associated with active systems are visible above the seafloor. Those associated with inactive systems are, however, likely to be buried below the seafloor, where they are protected from weathering^3^. Exploring methods that are applicable not only to visible but also to buried ore deposits should be chosen out of line-survey or mapping methods from the viewpoint of efficiency. Although such exploration methods are established in on-land environments, efficient methods for marine environments are not necessarily the same as those on land.

In on-land environments, the standard technique of exploring subsurface ore deposits is a remote sensing geophysical survey combined with geological information of the target area. The most effective methods for this purpose are electromagnetic methods with active sources^4^. These methods induce electric currents into the ground and measure their secondary responses. Such methods can reveal the two-dimensional or three-dimensional distribution of subsurface electrical conductivity, which is sensitive to the occurrence of metal-containing materials such as sulphide minerals. These methods, which are being developed for use in marine environments^5–8^, might also be used effectively in the ocean, but they are difficult to handle as tools for initial surveys without detailed information of the target area.

The passive self-potential method obtains a signal that is sensitive to the occurrence of hydrothermal ore deposits on land^9^. Buried ore deposits are detectable as a negative self-potential anomaly on the ground surface because of subsurface oxidation–reduction reactions occurring around sulphide minerals in the presence of a subsurface redox gradient, i.e. a geobattery^9^. This method originated in the nineteenth century as a tool for the exploration of buried ore deposits^10^, but it is not used frequently today as a method of on-land exploration. The self-potential is a derived quantity that is calculated from the measured data: potential differences are measured either by moving two electrodes alternately or by moving one of two electrodes with the other fixed; then the self-potential is calculated by adding measured potential differences among the measured points^10^. Although measurements themselves can be taken in a simple manner, merely by putting electrodes into shallow holes filled with clay minerals, the method presents shortcomings when used on land: some effort is needed to acquire a reliable dataset^10^; the method responds to various sources of electric current, such as fluid flow and temperature gradient^11^; and noise near the ground surface is occasionally high^12^. For these reasons, as a method of exploration in land environments, the self-potential method is minor compared to electromagnetic methods with active sources. Nevertheless, the self-potential method has been revived as a tool for imaging groundwater flow associated with volcanoes, geothermal systems, and contaminated aquifers combined with numerical modelling of fluid flow^10,13^. In these applications, because few means other than the self-potential method can detect subsurface fluid flow, the self-potential method in the imaging subsurface fluid flow overcomes the shortcomings described above^10,13^.

The self-potential method, although not the best method on land, is useful for exploring hydrothermal ore deposits in marine environments for the following reasons. First, electrodes in ambient seawater can measure *in situ* electrostatic potential referenced from a common electrode^14^ without special care that might be necessary for land environments, as discussed above. Thereby continuous measurements of a component of the electric field can be achieved using a pair of electrodes placed at a fixed distance and orientation. The electric field can be integrated along the survey line to yield the self-potential along it if the measured component of the electric field and the survey line are parallel. In this respect, the self-potential is also a derived quantity in marine environments.

Secondly, the effects of fluid flow or the streaming potential, which is several tens of millivolts in hydrothermal systems on land^10^, and those of noise for exploring ore deposits are estimated as sufficiently small in the ocean, as explained here. High-conductivity pore fluid in the marine environment decreases electrokinetic coupling (the magnitude of streaming potential per applied pressure gradient) by several orders of magnitude compared with that in the land environments^15^. Consequently, the streaming potential in the marine environments is expected to be considerably smaller than that in the land environments, assuming that the driving force of hydrothermal fluid flow is in the same order for both environments^16,17^. In addition, because the sign of the electrokinetic coupling coefficient is usually negative for geological materials including sulphides^18^, the self-potential signal is expected to be positive above fluid discharge. This tendency is opposite to that formed above an ore body by sub-seafloor oxidation–reduction reactions^9^. Towing at a constant speed produces a constant induced electric current, which is readily removable.

Thirdly, artificial electrical noise in the ocean (See Supplementary Table S1 for the present survey) is much less prevalent than on land. Examples for land environments are compiled in a recent monograph^10^. By virtue of these properties, towing two or more electrodes placed at different positions of a moving body measures the electric field along the dive track. Integration along the dive track obtains the self-potential. This characteristic is of particular importance for difficulties related to seafloor accessibility.

In marine environments, self-potential surveys of hydrothermal systems have been conducted since the 1970s^19–24^. Earlier reports have described anomalies of self-potential signals around hydrothermal deposits^19–23^. Later, Heinson *et al*.^24^ stated that the self-potential method is useful for detecting hydrothermal ore deposits below the seafloor. An active hydrothermal field was found based on a self-potential survey: Ashadze-2 on the mid-Atlantic Ridge^25^. Another survey revealed that the origin of the observed self-potential anomaly is oxidation–reduction reactions occurring between the discharged fluid and seawater^26^. Other recent reports have described the discovery of self-potential anomalies related to hydrothermal acticvities^27–29^. However, at present, the only quantitative analysis is determination of the source loci estimated from self-potential anomalies detected above a near-shore graphite deposit^24^. No quantitative analysis has been reported for an active hydrothermal site with sulphides.

We conducted a self-potential survey at a known hydrothermal field in the mid-Okinawa Trough, southern Japan^30^, using a deep-tow with an electrode array. We chose this hydrothermal field because it includes both active and inactive hydrothermal vents. Furthermore, the sulphide mound location is known^30^. We towed an electrode array at various altitudes from 5 to >50 m above the seafloor to verify the reliability of the observations. We analyse the observed data to show that geobattery model^9^ is valid at a first order. Our results confirm that the self-potential survey is an easy-to-perform method of detecting seafloor hydrothermal ore deposits of Kuroko type. The success of self-potential survey at the Kuroko-type ore deposit has important effects on future explorations because ore deposits of this type are regarded as promising targets for mining^31^.

## Materials and Methods

### Geological background

**Figure 1:**
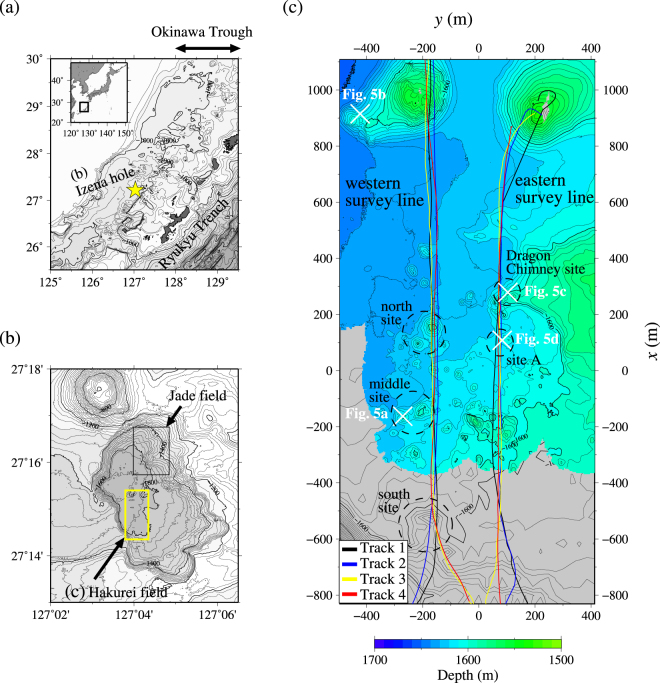
Maps of the target area. Drawn using freeware software (*Generic Mapping Tools Version 5*; http://gmt.soest.hawaii.edu/)^48^. (**a**) Map of the mid-Okinawa Trough with the inset portraying a regional map around Japan. The Izena hole location (shown in (**b**)) is denoted by a yellow solid star. The global dataset *ETOPO1* ^49^ is used to draw the bathymetry. (**b**) Map of the Izena hole with the yellow and black squares denoting the areas of Hakurei (shown in (**c**)) and Jade hydrothermal fields^32^. Bathymetric data were obtained during the MR13-E02 cruise (http://www.godac.jamstec.go.jp/darwin/cruise/mirai/mr13-e02_leg1/e) using a multi-narrow beam echo sounder (MBES) *SEABEAM3012* (sounding frequency was set to 12 kHz) equipped with *R/V Yokosuka*. (**c**) Map of the study area relative to the origin point (27°14.8′N, 127°04.1′E) with dive tracks of the two survey lines: black, blue, yellow, and red curves respectively denote Tracks 1–4. The dashed circles are locations of hydrothermal sites. The centres of the white crosses are locations at which the photographs presented in Fig. 5 were taken. The bathymetric data of the coloured area were obtained during the cruise YK14-17 (http://www.godac.jamstec.go.jp/darwin/cruise/yokosuka/yk14-17/e) using an MBES *SEABAT7125* equipped with the automated underwater vehicle *Urashima* (sounding frequency was set to 400 kHz). That of the grey area was the same as that used in (**b**) but shifted by 7.5 m in the shallow direction to match the YK14-17 data. The middle site is approximately 100 m west of the western survey line.

The target area of the present study, the Hakurei hydrothermal field, is located near the southwestern wall of a sedimented caldera floor, i.e. the Izena hole, located in the mid-Okinawa Trough^32^ (Fig. 1a,b). The caldera wall is 400 m high. Its typical water depth is 1600 m at the caldera floor.

A number of hydrothermal mounds have been observed in the Hakurei hydrothermal field^32^. Strong magnetic anomalies were observed around some of these hydrothermal mounds^33^, which consist of sulphide minerals of high magnetisation. Their mineral assemblage^34^ resembles that of Kuroko-type deposits characterised by Cu-Pb-Zn sulphides^35–37^. Sulphide minerals of drilling/recovered samples include an average 3.2 g/t of Au^38^. Deep-sea drilling conducted by the Japan Oil, Gas and Metals National Corp. (JOGMEC) revealed sulphides are distributed at two depths^38^, indicating that ore deposits in the Hakurei hydrothermal field are not limited to visible mound-like structures. Exposed sulphide mounds continue to approximately 30 m below the seafloor. Also, sulphide samples are recovered from approximately 50 m below the seafloor, indicating that sulphides are not limited to exposed hydrothermal mounds. The continuity between shallow sulphide mounds and deep sulphides is unknown. Along with these hydrothermal mounds, an active hydrothermal area has been reported: at the Dragon Chimney site, a large (>10 m high) chimney discharges fluids hotter than 300 °C^32^ (Fig. 1c).

### Observation

**Figure 2:**
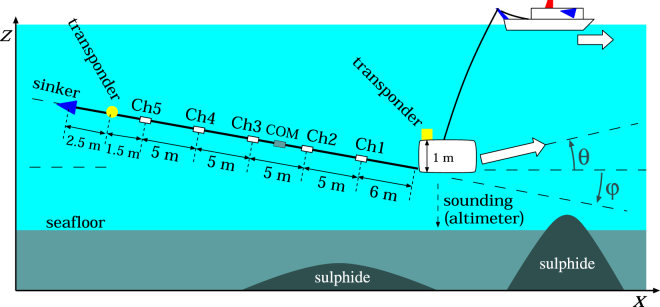
Schematic configuration of the self-potential measurement in the marine environment. An electrode array equipped with an elastic rod of 30-m-long is towed using a deep-tow apparatus. For the present study, five electrodes are used with 5 m separation. The rod has an angle *φ* relative to the horizon. The dive track has an angle *θ* relative to the horizon. This figure shows the case of negative *φ* and positive *θ*.

**Figure 3:**
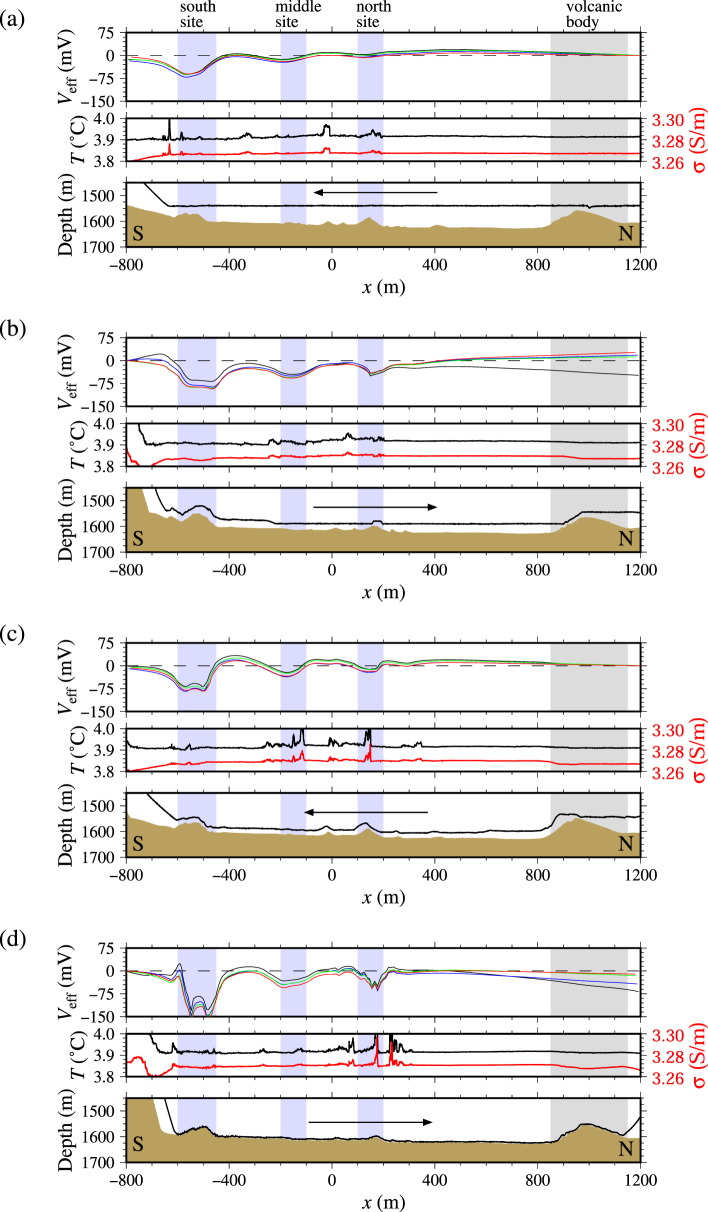
Results of the survey along the western survey line: (**a**–**d**) respectively correspond to Tracks 1–4. Shaded regions are of special interest (see remarks above the upper panel and the main text in detail). (Upper panel) Integrated effective self-potential along the dive track. The black, red, green, and blue curves respectively correspond to four pairs of 5-m-spacing electrodes from the deep-tow side. The measurement positions are given using the positions of the deep-tow and the tail of the rod (see Fig. 2). (Middle panel) Ambient seawater temperature (black curves) and electric conductivity (red curves) measured near the deep-tow. (Lower panel) Bathymetry obtained from the acoustic sounding and the pressure sensor equipped with the deep-tow (brown areas) and the positions of the deep-tow (black curves). The towing direction is indicated by the arrows.

**Figure 4:**
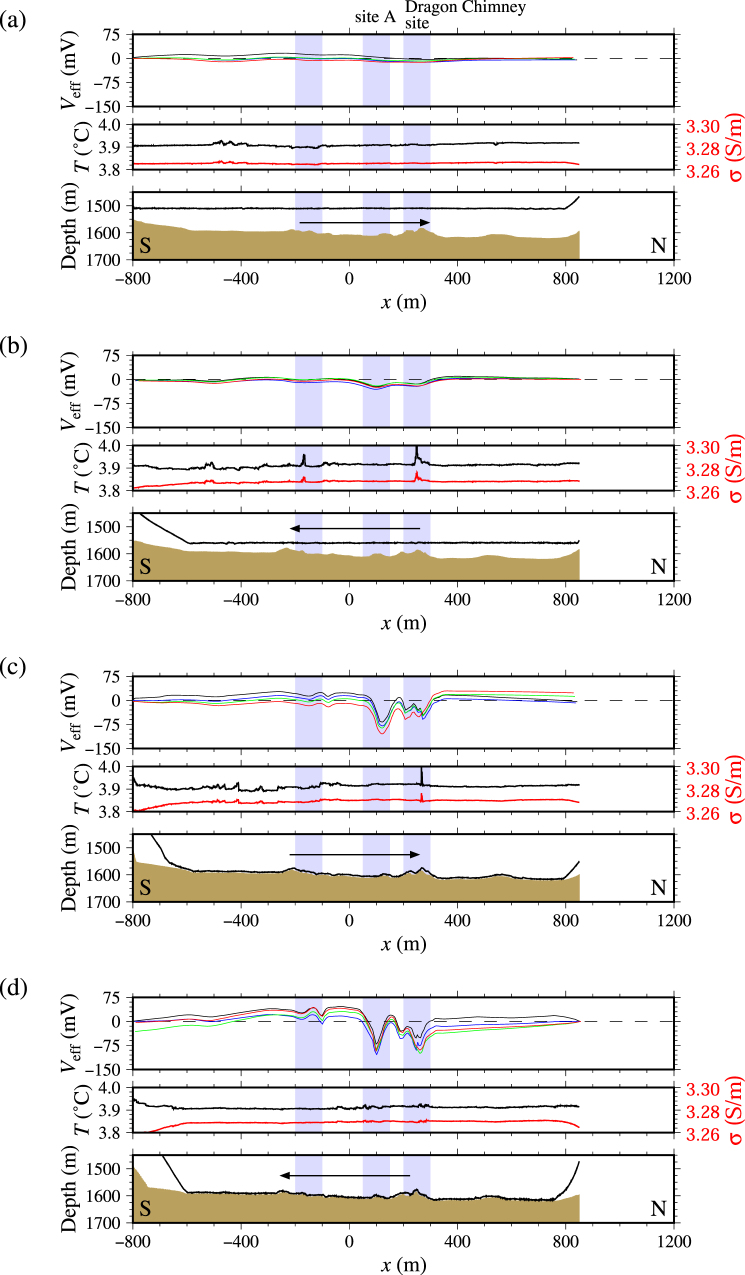
Results of the survey along the eastern survey line: (**a**–**d**) respectively corresponding to Tracks 1–4. Deep-tow heights of Tracks 3 and 4 ((**c**) and (**d**)) are the same, but the direction of towing is opposite. The notation is the same as that in Fig. 3.

We took continuous measurements of the electric field using a 30-m-long electrode array with a deep-tow handled by *R/V Yokosuka*. Five electrodes are mounted at equal intervals of 5 m along an elastic rod (Fig. 2; see *Supplementary document 1* for instrument details). We chose two survey lines (western and eastern) running approximately 2000 m north–south separated by about 250 m east–west (Figs 1c, 3 and 4). We towed the deep-tow at a constant speed of about 0.5 knots four times for each survey line to investigate the depth dependence of the self-potential signals. The deep-tow altitudes are 50, 30, 20, and 5 m in the western survey line and 50, 30, 5, and 5 m in the eastern survey line. To verify the reproducibility of the results, along the eastern survey line, we towed the deep-tow twice at the same altitude along the same dive track but in opposite directions.

During the survey, we also monitored the ambient seawater temperature and electrical conductivity near the deep-tow using a conductivity-temperature-depth (CTD) sensor to detect direct effects of discharged hydrothermal fluids. The deep-tow and the tail positions of the rod were monitored using acoustic transponders during the survey (Fig. 2). The latter is necessary to estimate each electrode’s position on the rod (*Supplementary document 1* explains the method used to determine the cable position.). Using an altimeter, the deep-tow altitude was also monitored during the survey. The bathymetry below the deep-tow dive track was calculated as the sum of the outputs of the depth meter and the altitude meter equipped with the deep-tow.

### Data analysis

The observed data are a time series of the electric potential of each electrode related to a common electrode (Fig. 2), the deep-tow position and altitude, and the cable angle along the dive track. Typical errors for these observed values are listed in Supplementary Table S1. We calculated the electric field using a pair of electrodes with the shortest sensor distance of 5 m because we found that the electric field is not constant along the 30-m-long rod, particularly when the deep-tow altitude is low (e.g. 5 m). In the present observation, the electric field direction was not parallel to the deep-tow dive track. Results showed that the tail of the rod was typically 10 m shallower than the deep-tow when towed at a constant water depth (Supplementary Figs S2 and S3). As a practical method, we integrate the observed electric field along the dive tracks to give the *effective* self-potential using the dive track. The self-potential obtained in this manner is regarded as *effective* because the directions of the electric field and the dive track are not always parallel (Fig. 2; see *Supplementary document* 1 for details).

To obtain the source locations corresponding to the observed self-potential signals and to ascertain whether a geobattery model proposed by Sato and Mooney^9^ is valid, we apply the probability tomography method^39–42^ as described in *Discussion*. Assuming the source as an electric current dipole, this method takes a cross-correlation between the electric field expected from a synthetic dipole and the observed electric field. We incorporate the directions of the measured electric field (*φ* in Fig. 2) and the survey track (*θ* in Fig. 2) into the analysis^42^. *Supplementary document 1* provides additional details. We did not use the integrated effective self-potential (upper panels in Figs 3 and 4) but used the electric field along the direction of the rod (Supplementary Fig. S1) for analyses because the directions of the electric field and the deep-tow track were not always in parallel during the survey. Furthermore, we ignored the contrast of electrical conductivity between seawater and sediment in this analysis. Synthetic tests conducted in the presence of a large electrical conductivity contrast (one order of magnitude) demonstrate that ignoring the contrast in the analysis negligibly affects the estimated source location within 10 m in most cases (*Supplementary document 2*). The overall probability of an electric current dipole (equation (S5) in *Supplementary document 1*) shows plausible locations for electric dipoles. Angles *φ* and *θ* have minor effects on the results in estimating the source location (*Supplementary document 3*).

## Results: Negative self-potential anomalies

**Figure 5:**
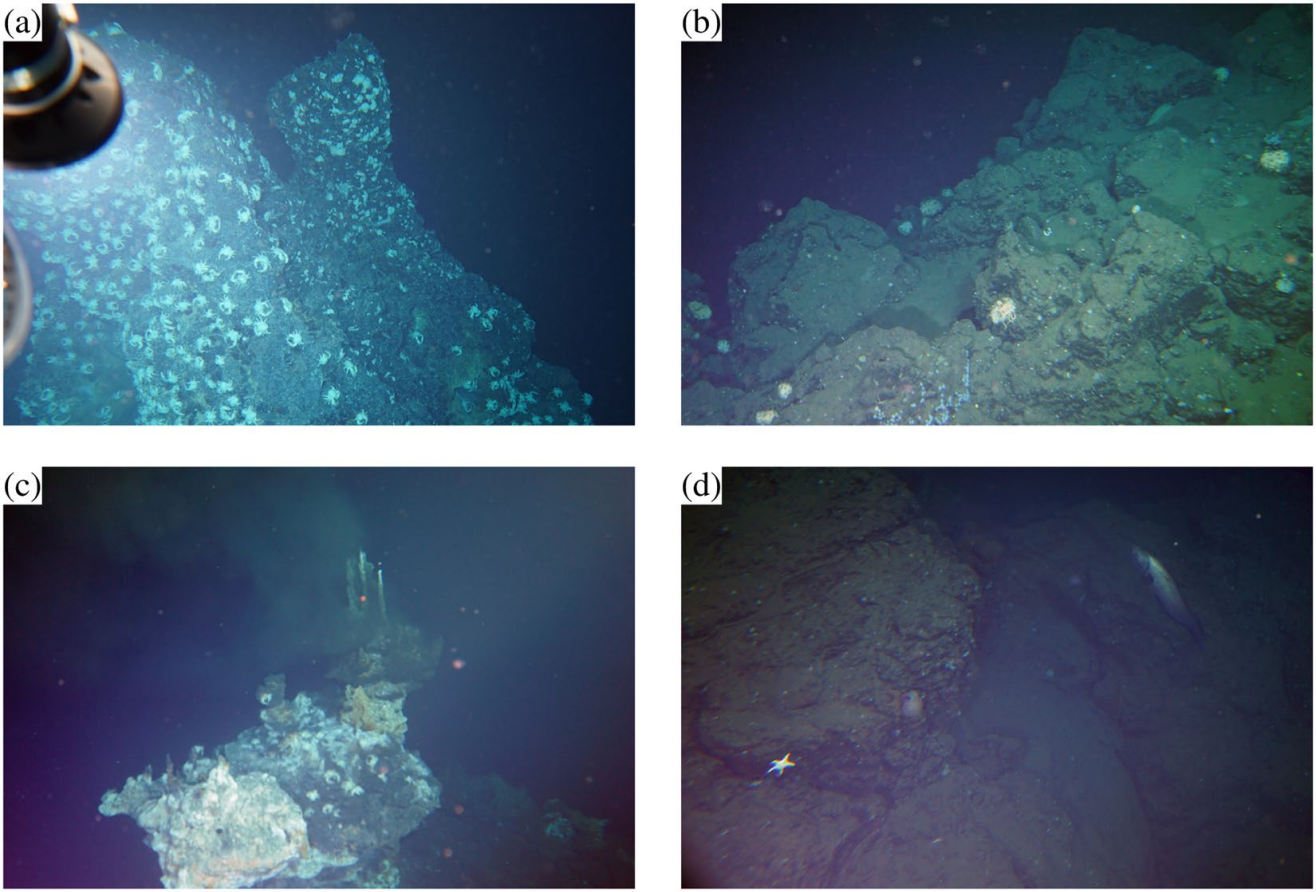
Photographs taken near the dive track of the self-potential survey. Taken from a crewed submersible, *Shinkai 6500*, during operation of the same cruise as that of the self-potential survey). See Fig. 1c for the location at which the photographs were taken. These photographs were taken by T. Kasaya and Y. Kawada. Western survey line: (**a**) active chimneys at the middle site (negative self-potential signal is observed nearby) and (**b**) a mountainside of a dacitic volcanic body with no self-potential signal detected. Eastern survey line: (**c**) a table-like sulphide mound at the Dragon Chimney site with negative self-potential signal and (**d**) site A with a larger-amplitude self-potential signal than that in (**c**) but no distinct hydrothermal activity or sulphide.

Along the western survey line, we have identified three major sites of active hydrothermal mounds, designated as the north, middle, and south sites (Fig. 1c). These are aligned north–south. We identified three major anomalies of negative self-potential at all four deep-tow altitudes (these tracks are designated as Tracks 1–4 in decreasing order of altitude; Fig. 1c and upper panels in Fig. 3; Supplementary Fig. S2 also shows the deep-tow altitude and the cable angle). These three anomalies respectively coincide with the south (*x* of approx. −550 m; with *x* being the relative north–south distance), the middle (*x* of approx. −200 m), and the north (*x* of approx. 150 m) sites. The active area of the middle site is approximately 100 m west of the dive track. The north and south sites are immediately below the dive track. These three sites all involve active hydrothermal chimneys (Fig. 5a is an example photograph of active chimneys taken at the middle site). We detected temperature and electrical conductivity anomalies near and around some of these sites (middle panels, Fig. 3). Typical temperature and electrical conductivity of seawater in the present survey area (approx. 1500 m deep) are, respectively, 3.9 °C and 3.27 S m^−1^. The temperature–conductivity anomalies and those of self-potential do not always mutually coincide. For instance, small-amplitude self-potential signals were obtained above a small mound at the north site (*x* of approx. 150 m), even with marked anomalies of temperature and electrical conductivity (Fig. 3d). However, the temperature anomalies always coincide with those of electrical conductivity. An example is apparent at *x* of approx. −50 m (middle panel, Fig. 3a). Anomalies of temperature and electrical conductivity are probably a direct effect of hydrothermal fluids. We detected no self-potential signal near a large seamount at *x* of approx. 1000 m (Fig. 1c and upper panels, Fig. 3), which has been identified as an igneous volcanic body of dacite^43^. We observed only bare igneous rock around this seamount, with no active vent or chimney (Fig. 5b).

Along the eastern survey line, we identified two major anomalies of negative self-potential at *x* of approx. 100 and 250 m and a minor anomaly of this kind at *x* of approx. −150 m (Fig. 1c and upper panels in Fig. 4; see also Fig. S3 for the deep-tow altitude and the angles of the deep-tow survey line and the electrode cable). The northernmost anomaly at *x* of approx. 250 m corresponds to an active hydrothermal site called the Dragon Chimney site^32^ (Fig. 1c), which consists of a table-like sulphide mound involving blacksmoker chimneys (Fig. 5c), where the discharge of hot fluids with temperatures of >300 °C have been observed. The anomaly found at *x* of approx. 100 m, which we call site A, does not involve active hydrothermal vents (Fig. 1c). This site has not been identified before with particular interests because we found no distinct hydrothermal activity or sulphide (Fig. 5d). We observed a temperature anomaly above the Dragon Chimney site accompanied with a conductivity anomaly (middle panels in Fig. 4). Temperature and conductivity anomalies were also observed above the minor self-potential anomalies at *x* of approximately −500, −150, and 250 m (middle panels in Fig. 4b). From our observations, site A exhibits the most intensive signal of the self-potential (*x* of approx. 100 m), but it does not involve active hydrothermal vents or temperature–conductivity anomalies (middle panels in Fig. 4).

Both western and eastern tow lines reveal higher self-potential anomalies as the amplitude of the deep-tow decreases. They show that short-wavelength signals are dominant (upper panels in Figs 3 and 4). Along the western line, the self-potential signal above the south site shows a single peak of 50 m above the seafloor (Fig. 3a,b), which is split into two peaks at lower altitudes (Fig. 3c,d). This characteristic is expected because the self-potential satisfies the Laplace equation outside of the source of the signals. The self-potential signal detected near the middle site (*x* of approx. −200 m) does not become larger when the altitude is decreased. This lack of change is probably related to a geometrical effect because the middle site is approx. 100 m to the west of the dive track (Fig. 1c). The distance between the source and the deep-tow does not become smaller when the altitude is decreased. Along the eastern line, two tracks with nearly equal altitude (approx. 5 m; Tracks 3 and 4) reveal self-potential signals with similar amplitudes indicating reproducibility of the signal (upper panels, Fig. 4c,d). However, the individual electrode pairs show somewhat different amplitude along the tracks (Supplementary Fig. S1).

## Discussion

### Validation of the geobattery model

**Figure 6:**
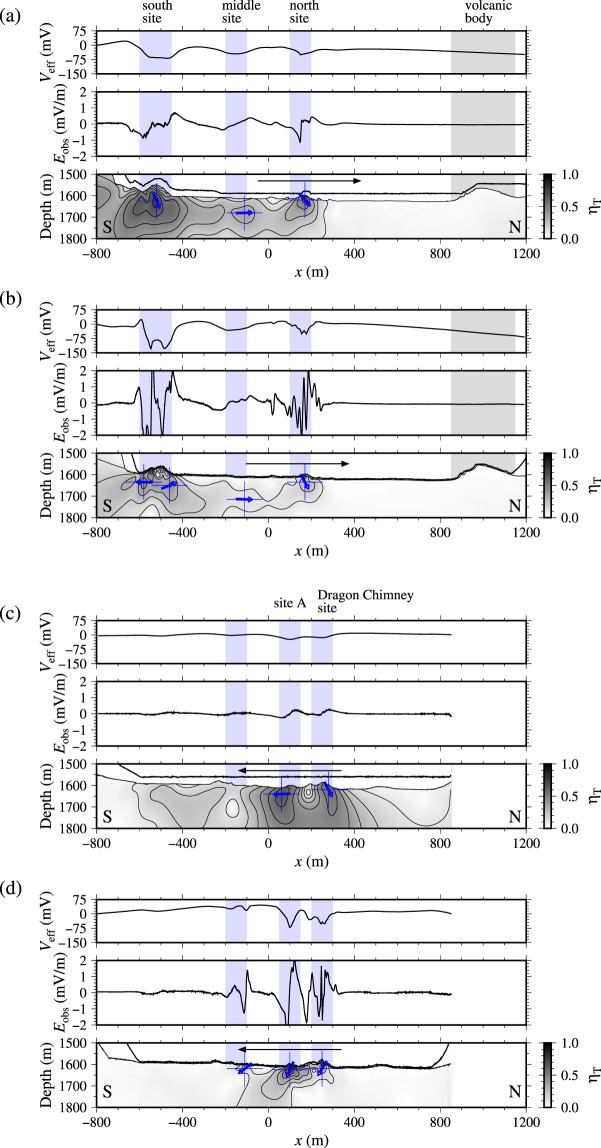
Results of exploring the source of the observed self-potential anomalies using probability tomography: (**a**) and (**b**) respectively show Tracks 2 and 4 of the western survey line (corresponding to Fig. 3b,d); (**c**) and (**d**) respectively show Tracks 2 and 4 of the eastern survey line (corresponding to Fig. 4b,d). See Supplementary Fig. S4 for other Tracks. (Upper panel) The effective self-potential calculated from the nearest dipole pair to the deep-tow (i.e. electrode channels 1 and 2 in Fig. 2). (Middle panel) Observed electric field. (Lower panel) The overall probability of an electric current dipole defined by equation (S5) in *Supplementary document 1*. The colour scale is shown to the right of each panel. The contour interval is 0.1. Blue arrow corresponds to the polarisation direction of dipoles, where the occurrence probability takes the local maximum (Supplementary Table S2 presents the estimated location and polarisation).

We apply the probability tomography method to ascertain whether the geobattery model proposed by Sato and Mooney^9^ is valid (Fig. 6 and Supplementary Fig. S4). In the model, a buried ore body with high electrical conductivity crossing the vertical redox gradient generates electric current to form a negative self-potential anomaly above the body^9,41^. Vertical electric dipoles with downward polarisation are imaged below the seafloor if the conditions of Sato and Mooney’s are perfectly realised.

Here we present only those results obtained from a pair of electrodes nearest to the deep-tow separated by 5 m (i.e. electrode channels 1 and 2, Fig. 2). The observed electric field used for analyses and the calculated effective self-potential are shown for reference in the middle and upper panels in Fig. 6 and Supplementary Fig. S4. Other combinations of electrode pairs provide fundamentally equivalent electric fields if the electrode positions are given properly using the positions of the deep-tow and the tail of the rod (Supplementary Fig. S1). The occurrence probability is calculated with intervals of 10 m in the horizontal direction and 5 m in the vertical direction.

The probability tomography analysis yields the overall occurrence probability of electric current dipoles (defined by equation (S5) in *Supplementary document 1*) with downward subvertical to horizontal polarisation located below the seafloor (blue arrows in lower panels of Fig. 6 and Supplementary Fig. S4 correspond to the polarisation direction defined by equation (S6) in *Supplementary document 1*; See Supplementary Table S2 for the estimated source depth; See Supplementary Figs S5 and S6 for the occurrence of horizontal and vertical dipoles defined by equation (S2) in *Supplementary document 1*). Along the western survey line, two regions have high probability of having electric dipoles (lower panels in Fig. 6a,b). They respectively correspond to the south and north sites. Two horizontal dipoles facing each other are given at the south site by a low-altitude survey (Fig. 6b); the self-potential signal formed by two horizontal dipoles of this kind is nearly identical to that formed by a single downward dipole. The middle site, which is offset of approx. 100 m of the survey line, is poorly resolved (a horizontal dipole is obtained), although there exists a distinctive negative self-potential anomaly. In this site, only one of two horizontal dipoles facing each other might be detected. The probability becomes diffusive for high altitude surveys because of the characteristics of potential fields.

Along the eastern survey line, two regions are detected with a high probability of electric dipoles with downward subvertical polarisation. They respectively correspond to the Dragon Chimney site and site A (lower panels in Fig. 6c,d). Shallower depths are expected here than those found below the western survey line. The horizontal location of the sources is well determined during high deep-tow altitude surveys. The source depth, however, is poorly obtained from these surveys (Fig. 6c). For a low altitude survey, another dipolar source is resolved at *x* of approx. −100 m (Fig. 6d).

Subvertical dipoles are imaged below some of the observed negative self-potential anomalies (the Dragon Chimney site is an example; Fig. 6c,d), which is consistent with the geobattery model^9^. On the other hand, pairs of horizontal dipoles facing each other are also observed below the negative self-potential anomalies (the south site is one example; Fig. 6b). The present imaging method cannot completely distinguish signals of these two types that form similar self-potential signals. Such pairs of horizontal dipoles facing each other might not be inconsistent with the geobattery model. However, interpretations using this imaging method demand care. The method should not be used to analyse detailed features of buried ore bodies.

### Source location of the self-potential signals

A geobattery model driven by the subsurface redox gradient proposed by Sato and Mooney^9^ might work below the hydrothermal ore bodies as described above. However, there is another mechanism that results in anomalies of the self-potential: a geobattery working above the seafloor driven by the redox potentials between the fluids in a hydrothermal plume and its surrounding seawater. Here we consider these two mechanisms separately.

Let us first consider Sato and Mooney’s deep geobattery model^9^, in which a dipole with downward polarisation results in a negative self-potential anomaly above the seafloor. The occurrence of subvertical downward dipoles is consistent with Sato and Mooney’s model, and the occurrence of a pair of horizontal dipoles facing each other does not contradict their model. Furthermore, importantly, as explained in the previous subsection, we estimated the source locations below the seafloor as the model predicts (Fig. 6). Buried sources were found based on the fact that the self-potential signal intensity increases concomitantly with decreasing deep-tow altitude (Figs 3 and 4). The next step is to examine whether buried current sources are expected.

For the deep geobattery model to work, the difference in redox potential is required at the depth of source depth of the self-potential anomaly. In a land environment, a water table crossing an ore body behaves as a reduction–oxidation boundary^9^. In a marine environment, the presence of fluid circulation around an ore body introduces oxic seawater, which intensifies the oxidation–reduction boundary below the seafloor. At the Izena hydrothermal field, results of a geothermal study suggest that the heat source depth is approx. 200 m below the seafloor at the Jade hydrothermal field^44^, approx. 2 km northeast of the Hakurei hydrothermal field^32^ (Fig. 1b). In addition, the origin of high-temperature hydrothermal fluids discharging from the Jade and Hakurei fields is geochemically the same^32^: both discharges are influenced by the surrounding sediments. Therefore, we expect the heat source and hydrothermal circulation of the Hakurei hydrothermal field to be approximately 200 m deep^32^. The source depth of the electrical dipole can be as deep as the heat source. A deep redox front of such a kind might be possible for sediment-hosted hydrothermal systems other than Kuroko-type, which we have studied, because sediment-hosted hydrothermal systems tend to have fluid circulation within the sediment^45^. Without the presence of sediments, a large sulphide mound, inside of which fluid circulation takes place, might accompany deep fluid circulation, producing the self-potential anomaly. An example of this case is the TAG hydrothermal mound at the mid-Atlantic Ridge^22^.

Let us move to consider another possibility, the geobattery working above the seafloor. A simultaneous survey of the electric field and redox potential across a hydrothermal plume indicates that the occurrence of these anomalies is related^26^. That survey found that both observed anomalies are direct consequences of reduced hydrothermal fluids in a plume, produced by chemical reactions between reduced fluids in a plume and the surrounding oxic seawater. A similar model is proposed for the land environment^46^, in which subsurface heterogeneities in redox potential simply cause the emergence of self-potential anomalies without the presence of an ore body. However, little discussion has been made of the theoretical aspect of redox condition within a hydrothermal plume.

A geobattery model for a reduced contaminant plume^47^ in the land environment provides some insights to this problem: the resultant self-potential is proportional to the solid angle of the body viewed from the observation point as well as the redox difference between the body and its surrounding. According to this model, an isolated fluid body having a different redox potential from its surroundings shows no self-potential anomaly outside it. Consequently, for this ideal case in which the redox contrast is constant, no self-potential anomaly is expected outside of a reduced body^47^. As another indication of this model, it predicts the existence of positive anomalies inside a reduced fluid body, which is confirmed in the land environment^47^. We might also expect the existence of positive self-potential anomaly inside a reduced hydrothermal plume in the marine environment. For more general cases in which mixing between hydrothermal fluid and seawater forms a diffuse contrast in the redox potential, gradual self-potential anomaly might be expected around a plume, but its sign has the same as the ideal case, i.e. positive self-potential anomalies inside the plume. In addition, the geobattery working above the seafloor has the current source above the seafloor. These predictions are inconsistent with our results (Fig. 6).

Although the self-potential anomalies observed in the present study might have their origin below the seafloor, more precise inversion analyses with a detailed electric conductivity structure can provide better estimation. For example, our estimated depth of the order of several tens of metres is not very accurate. Future surveys might reveal the redox potential and the self-potential simultaneously, which can specify the depth of self-potential sources and which can quantify the effect of hydrothermal fluids on the self-potential. In addition, the distribution of electrical conductivity might help us to estimate the burial depth.

### Self-potential method as a tool for exploring the potential of hydrothermal ore deposits

The self-potential method is a powerful tool for initial surveys to explore seafloor hydrothermal deposits in terms of the three characteristics. First, the self-potential method can detect the hydrothermal mound location. We observed negative self-potential anomalies above three known hydrothermal mound sites (north, middle, and south sites in the Hakurei hydrothermal field^32^; Figs 1c and 5a) and a site of active hydrothermal vents (Dragon Chimney site; Fig. 5c). By contrast, no self-potential signal was found above a dacitic volcanic mound (Fig. 5b). These exposed mound-like structures are classifiable by self-potential measurements as having either hydrothermal (e.g. north, middle, and south sites) or non-hydrothermal (e.g. dacitic volcano) origin. This characteristic is particularly useful for initial surveys that explore vast areas. If a precise bathymetry map of a target area is used on an occasion of a newly projected survey, then a self-potential survey might be sufficient to categorise any mound-like structure. For that reason, volcanic mounds that are useless for exploration would be excluded as targets. Extinct hydrothermal structures with mineralisation can be detected from a self-potential survey if the redox gradient crosses the ore body and if the geobattery of Sato and Mooney^9^ works. Future exploration including deep-sea drillings might confirm our prediction.

Secondly, this method might detect hydrothermal ore deposits that are buried below the seafloor. We observed negative self-potential signals above the flat seafloor without apparent hydrothermal activity along the eastern survey line (e.g. site A; Fig. 5d). It is particularly interesting that the self-potential anomaly above the site is larger than that above the Dragon Chimney site with hydrothermal vents (Figs 1c and 5c). We anticipate that ore deposits are buried below site A (Figs 1c and 5d), where there is no apparent hydrothermal activity or hydrothermal mound above the seafloor. However, the possibility exists that hydrothermal activities are overlooked during observations. More detailed observations must be conducted to verify this characteristic.

### Limitations and future perspectives of the proposed method

The self-potential method, of course, has limitations from a scientific perspective. First, the self-potential method itself does not identify the mechanism for the self-potential signals detected below the seafloor. The existence of electric current dipoles can be regarded as a result of oxidation–reduction reactions occurring below the seafloor^9^ because the streaming potential is expected to have a minor effect in the ocean, as discussed above. Oxidation–reduction reactions around ore bodies embedded in the sediment accumulate negative (positive) electric charges in the shallower (deeper) part because of the surrounding redox gradient (upward oxic)^9^. The resultant spatial imbalances of electric charges around these ore bodies can generally be approximated by electric current dipoles^41^. Revealing the origin of electric current dipoles is the next step of prospecting. Simultaneous measurements of the self-potential and redox potential are of importance to distinguish the origins of self-potential anomalies from oxidation–reduction reactions occurring below the seafloor and discharged hydrothermal fluids. In addition, investigating mineral assemblages and pore water chemistry as well as modelling fluid flow with chemical reactions might provide information related to redox reactions combined with fluid flow occurring below the seafloor. Such knowledge is expected to provide necessary information to improve estimation of the mechanism that generates electric current dipoles. The progress of such research is the same for the self-potential method as a tool of imaging fluid flow in land environments. The self-potential method in land environments has been revived to produce a quantitative method by combining numerical modelling of fluid flows^13^.

Secondly, three-dimensionality of the distribution of ore deposits should be considered during exploration. Acquiring data along nearly straight survey lines, we analyse the data in a two-dimensional system in this study. However, as presented in Fig. 6, the data reflect three-dimensionality near the middle site (*x* of approx. −200 m) of the western survey line, for example, where the mounds are offset approx. 100 m westward of the survey line (Fig. 1c). The source location can be estimated practically if precise bathymetry is obtainable in advance: in the present case, the source is located west of the survey line, where mound-like structures are observed. To detect the actual source location precisely, more than two survey lines must be located across the source because of the characteristics of potential problems. Alternatively, surveys with several altitudes along the same survey line cause similar effects. In this latter case, the response of the source intensity depends on the offset along the survey line. However, one cannot specify whether the source is located at the left side or the right side of the survey line.

Finally, given the minor limitations described above, we conclude that the self-potential method is a useful tool for initial surveys designed to detect hydrothermal ore deposits in the marine environment. This easy-to-perform method, merely towing an electrode array several tens of metres above the seafloor without complicated analysis, is applicable to explore the potential occurrence of visual (exposed) or even completely buried hydrothermal ore deposits. It is surprising that such a classical method originating in the nineteenth century^10^ remains among the fastest and easiest methods of detecting signals from seafloor hydrothermal ore deposits. The success of the self-potential survey in the marine environment can redefine geophysical exploration to locate buried hydrothermal ore deposits.

## Conclusions

We observed negative self-potential signals above Kuroko-type hydrothermal sulphide mounds in the Hakurei hydrothermal field of the Okinawa Trough, southern Japan. Results confirmed that the self-potential method can detect the presence of ore deposits using only a towed electrode array installed on a deep-tow, even without complicated analysis (Figs 3 and 4). This characteristic is important for initial surveys, during which seafloor hydrothermal ore deposits must be identified from a state having little information related to the target area. Results suggest that the origin of the observed self-potential anomalies comes below the seafloor. If that is true, then completely buried ore deposits are detectable.

### Data Availability

The datasets generated during and/or analysed during the current study are not publicly available due to its proprietary nature but are available from the corresponding author on reasonable request.

## Electronic supplementary material

Supplementary Information
