# Supplementary material for: Marine self-potential survey for exploring seafloor hydrothermal ore deposits
Source: Sci Rep. 2017 Oct 19;7:13552. doi: 10.1038/s41598-017-13920-0 (PMC5648760; doi:10.1038/s41598-017-13920-0)
Supplement: Supplementary file 1 — Supplementary Information [file 41598_2017_13920_MOESM1_ESM.pdf]

Supplementary Information

Marine self-potential survey for exploring seafloor hydrothermal ore deposits

Yoshifumi Kawada[1, 2 *] & Takafumi Kasaya[3, 1]

1. Project Team for Development of New-generation Research Protocol for Submarine Resources, Japan Agency for Marine-Earth Science and Technology, 2-15 Natsushima-cho, Yokosuka 237-0061, Japan
2. International Research Institute of Disaster Science, Tohoku University, 468-1 Aoba, Aoba-ku, Sendai 980-0845, Japan
3. Research and Development Center for Earthquake and Tsunami, Japan Agency for Marine–Earth Science and Technology, 2-15 Natsushima-cho, Yokosuka 237-0061, Japan

*** Corresponding author:** kawada@irides.tohoku.ac.jp

Supplementary information includes Figs. S1–S6 and Tables S1–S2 cited directly in the main text, with three supplementary documents.

Supplementary figures cited in the manuscript.

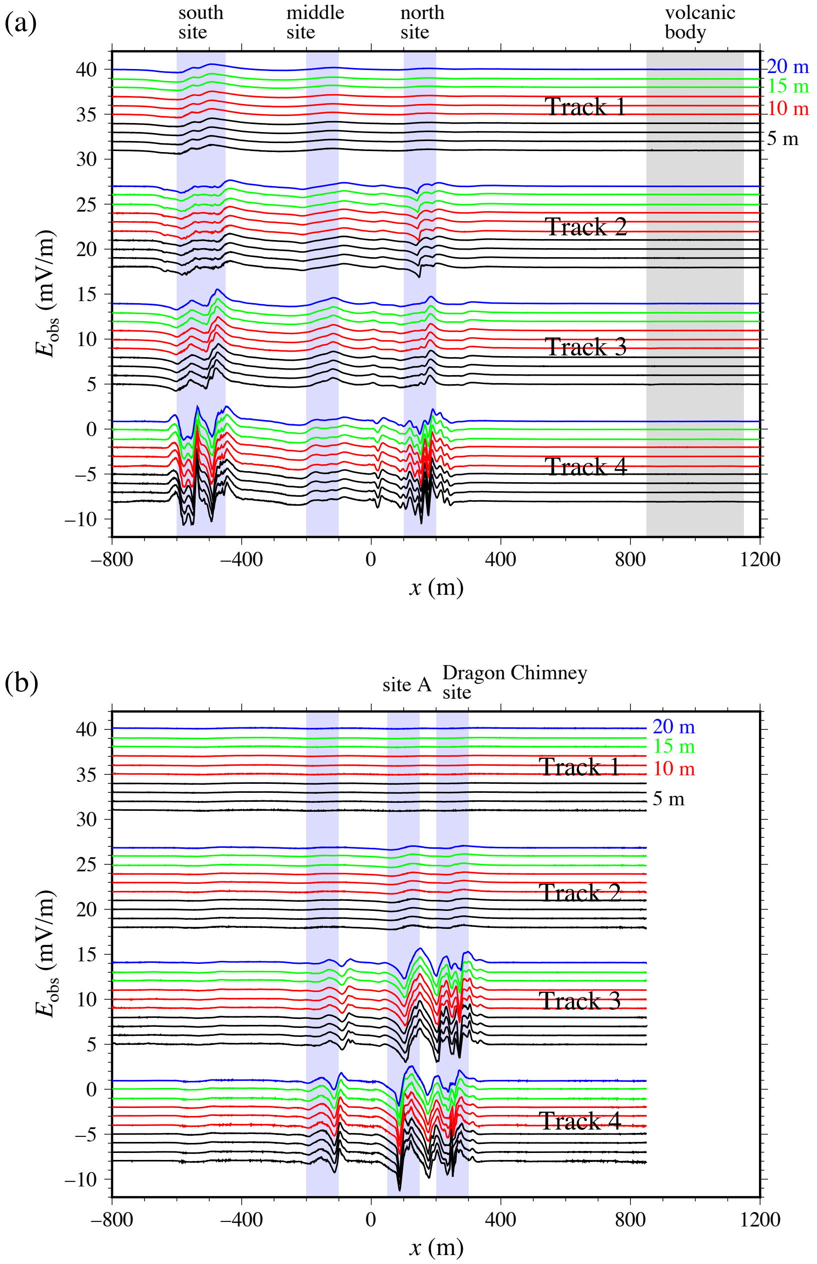

Figure S1. Electric field obtained during the survey: (a) and (b) respectively portray western and eastern survey data. Data are shifted vertically for ease of viewing. Black, red, green, and blue curves respectively correspond to pairs of 5, 10, 15, and 20-m-spacing electrodes. For colours other than blue, deep-tow side pairs are shown at the lower side.

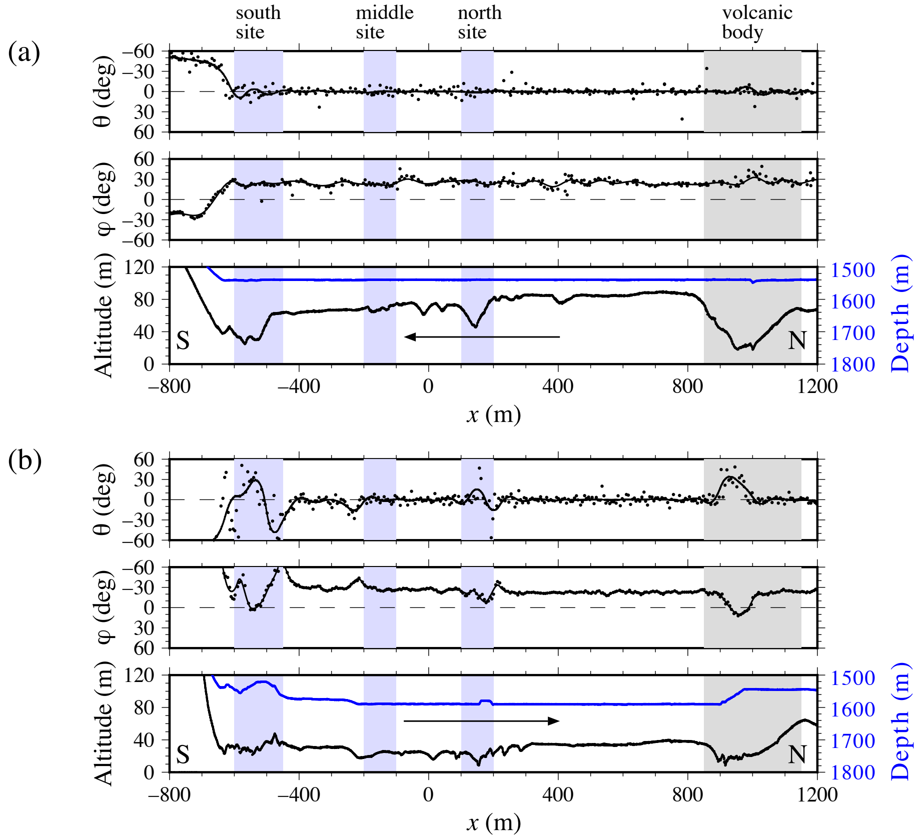

Figure S2. Deep-tow and electrode cable positioning: (a)–(d) are Tracks 1–4 of the western survey line. (Upper and middle panels) Slope angles of the deep-tow dive track (θ) and the electrode cable (φ). Some of the vertical axes are inverted so that the upper half of these figures represents angles above the horizon. (Fig. 2 presents definitions of these angles). Dots show the measured data. Curves present results of least-squares fitting. (Lower panel) Deep-tow altitude (black curves with the left axis) and depth (blue curves with the right axis). Arrows indicate the towing direction. Shaded areas are of special interest (Details are presented in *Results*.).

(c)

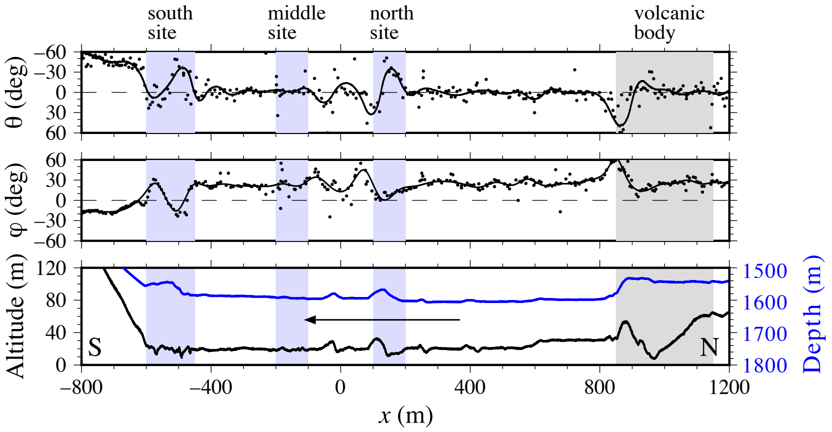

(d)

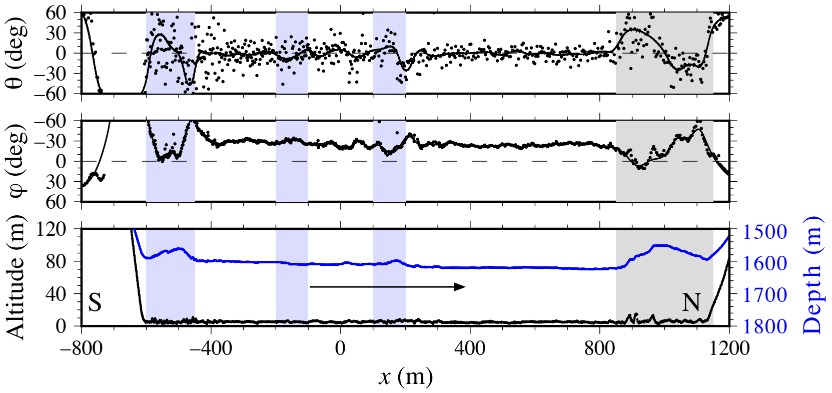

Figure S2. (Continued)

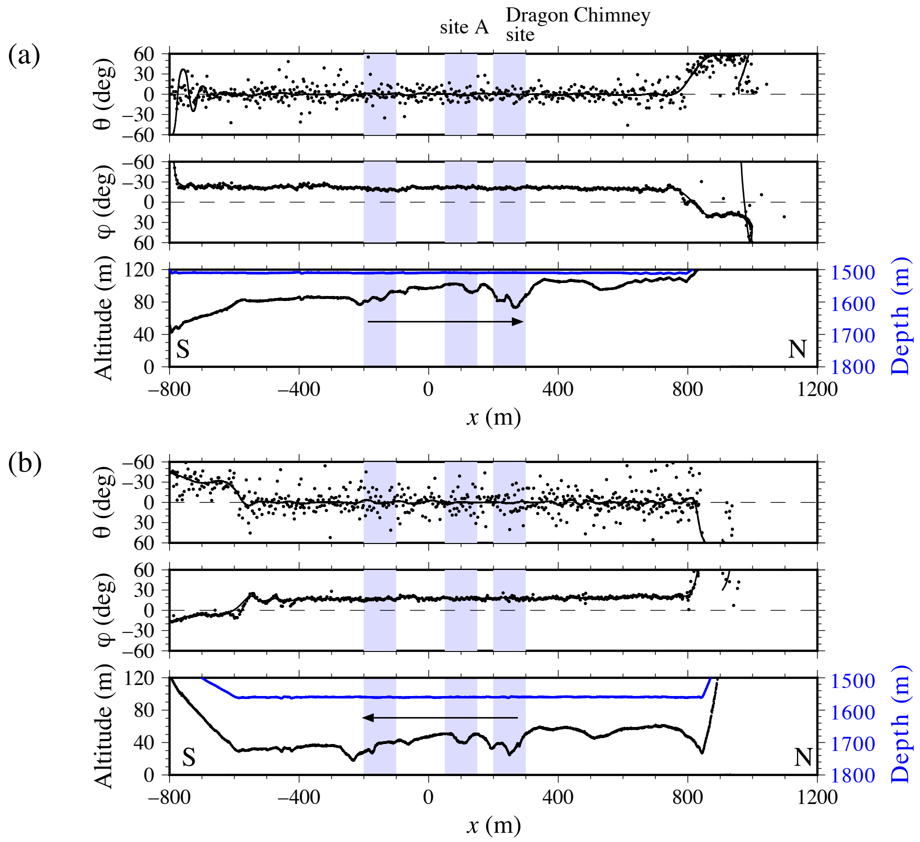

Figure S3. Deep-tow and electrode cable positioning: (a)–(d) show Tracks 1–4 of the eastern survey line. (Upper and Middle panels) Slope angles of the deep-tow dive track (θ) and the electrode cable (φ). (Fig. 2 gives definitions of these angles). (Lower panel) Deep-tow altitude (black curves) and depth (blue curves). The notation is identical to that in Fig. S2.

(c)

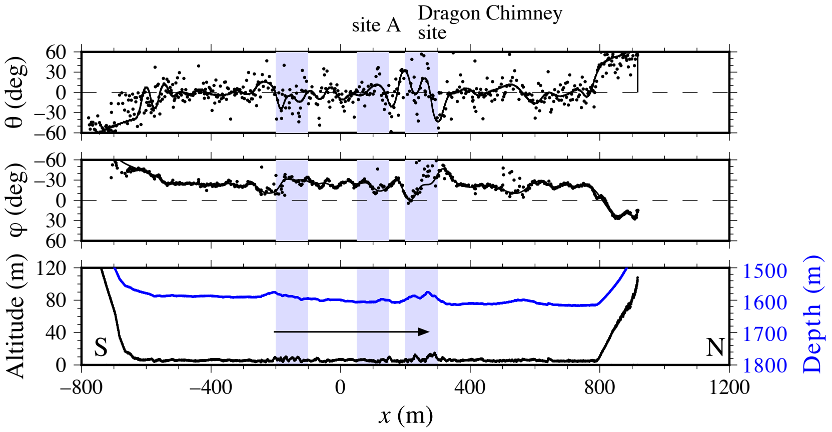

(d)

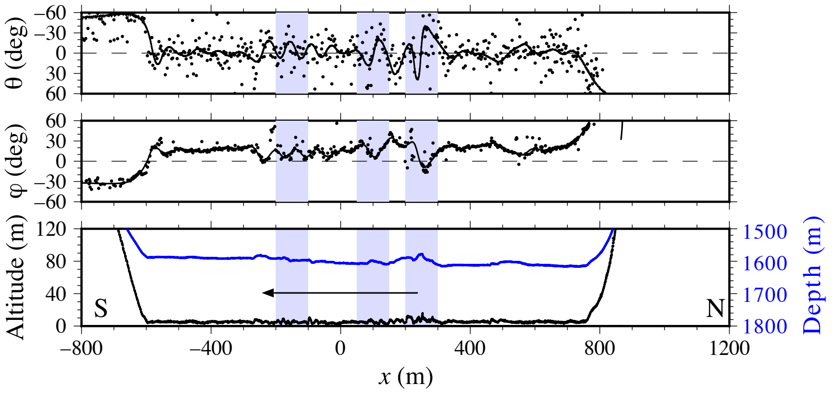

Figure S3. (Continued)

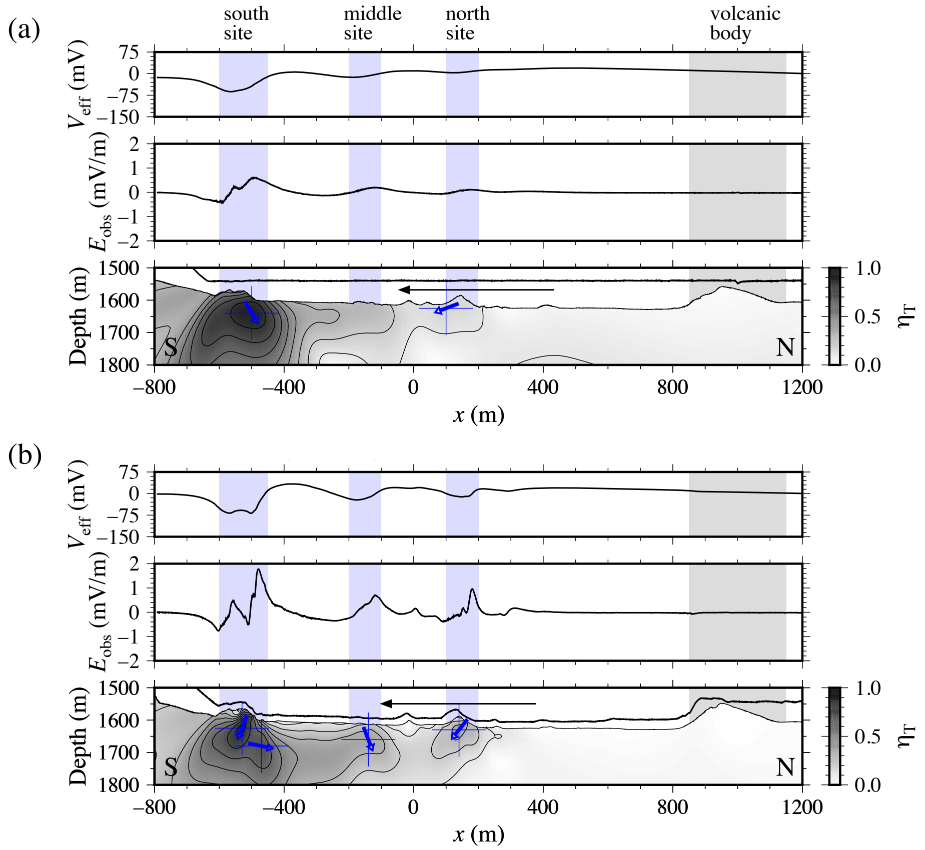

Figure S4. Results from exploration of the source of the observed self-potential anomalies, which complement Fig. 6: (a) and (b) respectively show Tracks 1 and 3 of the western survey line; (c) and (d) respectively show Tracks 1 and 3 of the eastern survey line. (Upper panel) Effective self-potential obtained from the nearest dipole pair to the deep-tow (electrode channels 1 and 2 in Fig. 2). (Middle panel) Observed electric field. (Lower panel) Overall occurrence probability of an electric current dipole; colour scales are shown at the right of respective panels. The contour interval is 0.1. Black curves show the deep-tow dive track. The blue arrow indicates the polarisation direction of dipoles, where the occurrence probability takes the local maximum. *Supplementary* Table S2 shows the estimated location and polarisation.

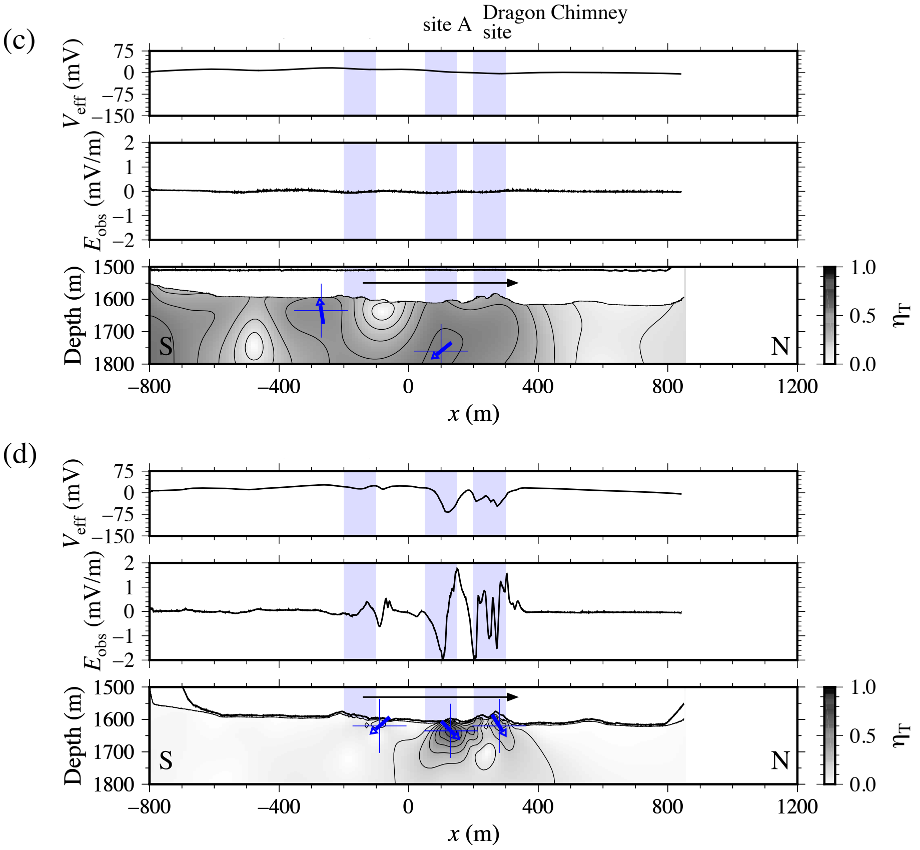

Figure S4. (Continued)

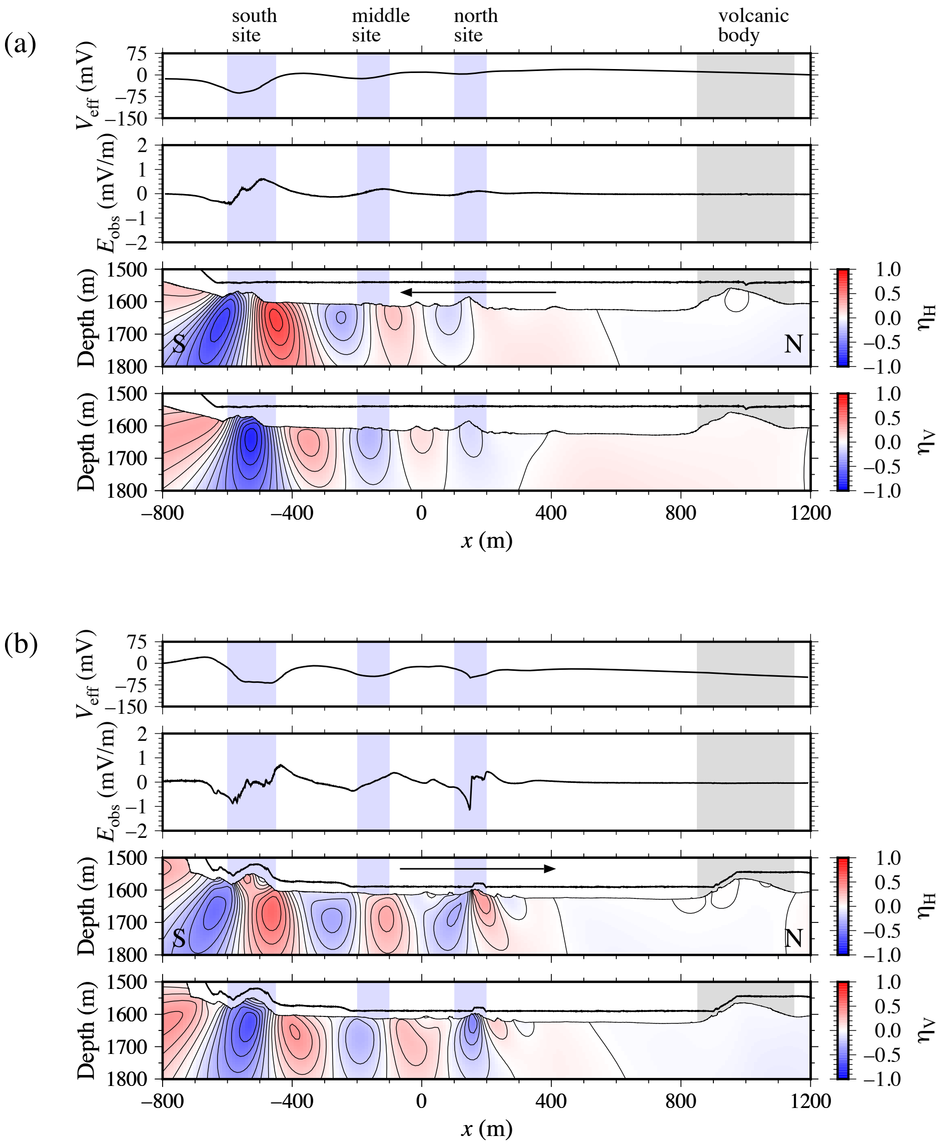

Figure S5. Results from exploration of the source of observed self-potential anomalies, which complement Figs. 6 and S4: (a)–(d) respectively show Tracks 1–4 of the eastern survey line. (Upper panel) Effective self-potential calculated from the electric field. (Second panel) Observed electric field used for calculating the probability. (Third panel) Occurrence probability of a horizontal dipole defined by equations (S2)–(S4) in *Supplementary document 1*. Negative (blue) and positive (red) colours respectively show rightward and leftward polarisation. The contour interval is 0.1. (Lower panel) Occurrence probability of a vertical dipole (equations (S2)–(S4) in *Supplementary document 1*). Negative (blue) and positive (red) colours respectively denote downward

and upward polarisation. The contour interval is 0.1. Black curves show the deep-tow dive track.

(c)

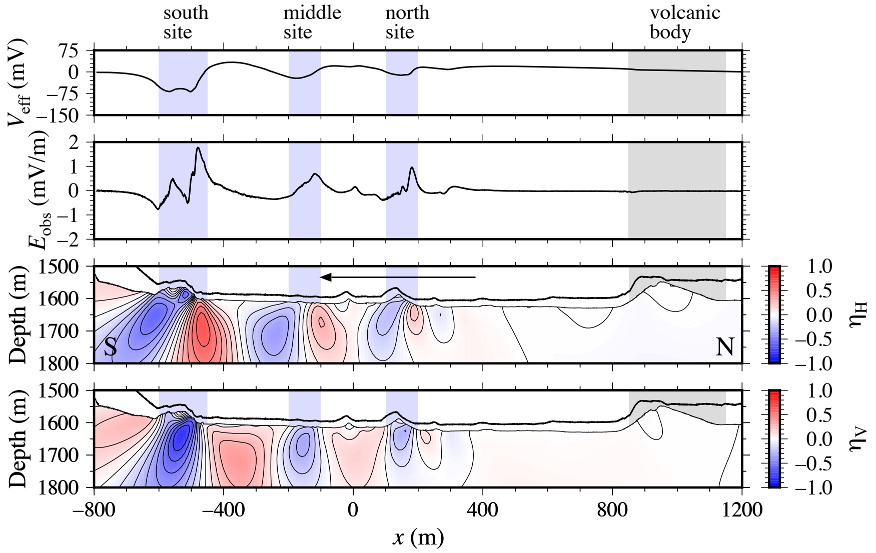

(d)

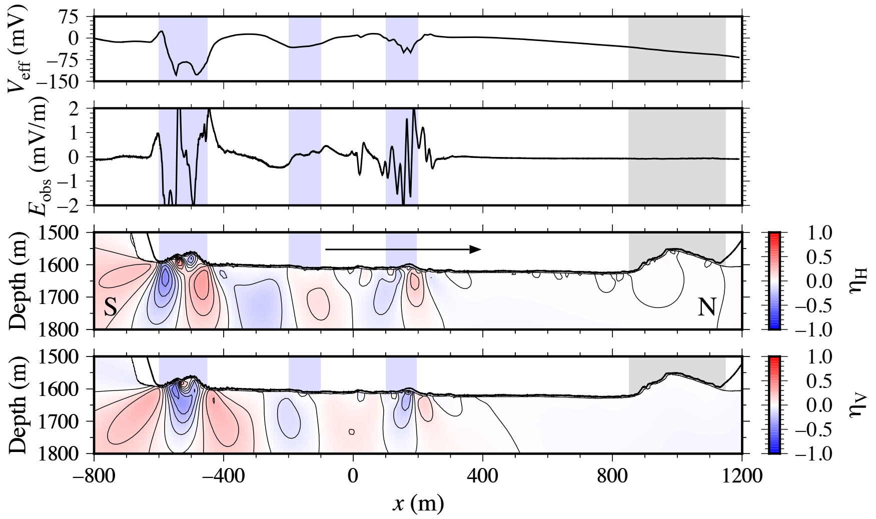

Figure S5. (Continued)

(a)

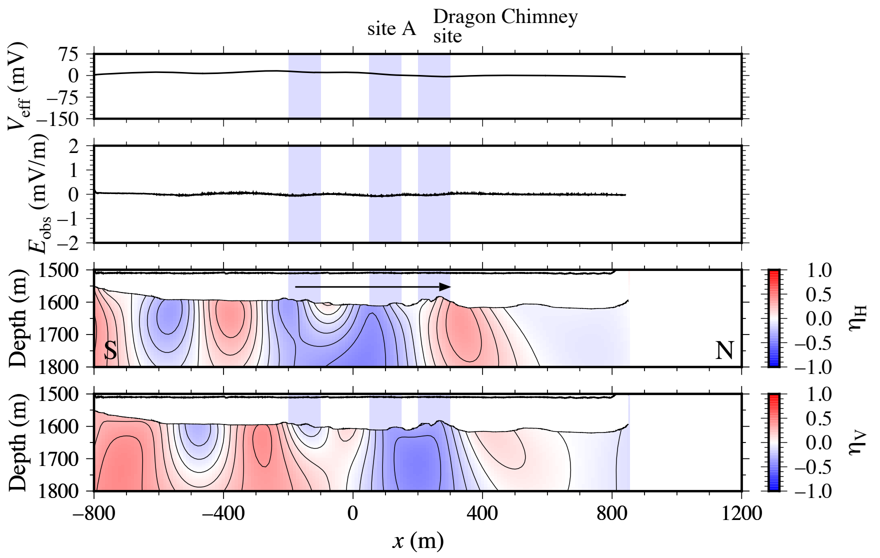

(b)

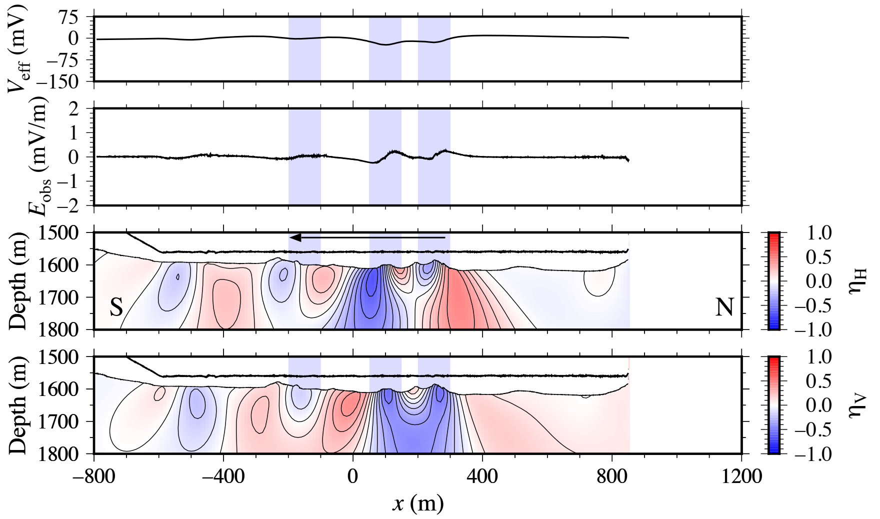

Figure S6. Results of exploring the source of the observed self-potential anomalies, which complement Figs. 6 and S4: (a)–(d) respectively depict Tracks 1–4 of the western survey line. (Upper panel) Effective self-potential calculated from the electric field. (Second panel) Observed electric field. (Third panel) Occurrence probability of a horizontal dipole. The contour interval is 0.1. (Lower panel) Occurrence probability of a vertical dipole. The contour interval is 0.1. The notation is identical to that in Fig. S5.

(c)

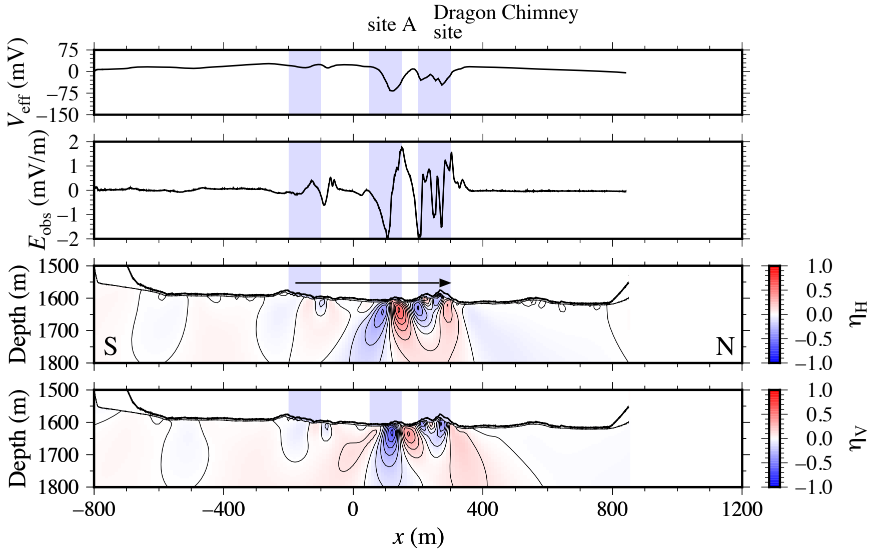

(d)

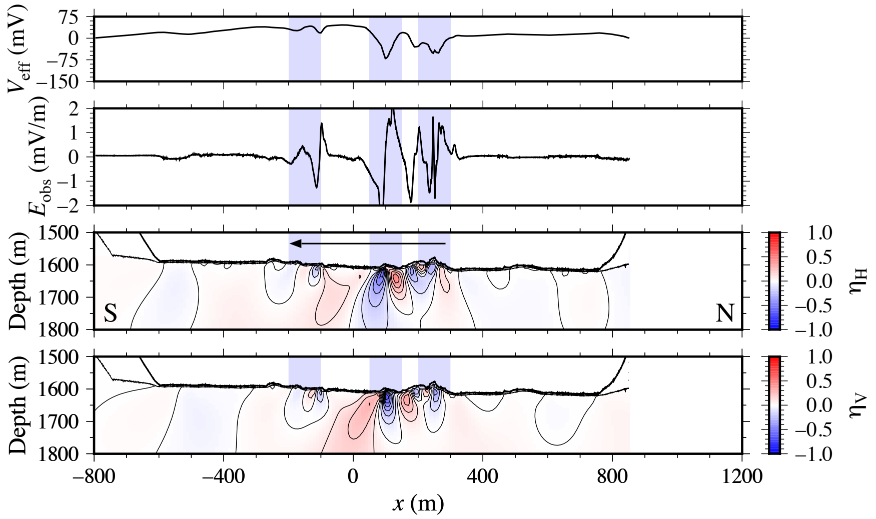

Figure S6. (Continued)

Table S1. Typical standard deviations of observed values during equal-altitude surveys

Value and Unit	Standard deviation	Remarks
Electric field (μV/m)	< 5	< 2 in most cases
Transponder, x and y (m)	< 20	< 10 in most cases
Transponder, z (m)	< 5	
Depth-meter and altimeter (m)	< 2	< 1 in most cases
Cable angle, φ (deg)	< 10	< 5 in most cases
Towing angle, θ (deg)	< 20	< 10 in most cases

Table S2. Depth and polarisation of the detected dipoles

Site Name	Dive track	*x* (m)	*z* (m)	Polarisation* (deg)
South site	1	–500	1640	–60
	2	–520	1630	–72
	3	–530	1625	–111
	3	–470	1625	–12
	4	–580	1635	178
	4	–460	1650	22
Middle site	1	n/a		
	2	–110	1680	2
	3	–140	1660	–69
	4	–110	1715	–2
North site	1	100	1625	–159
	2	170	1620	–48
	3	140	1620	–129
	4	170	1635	–62
Dragon	1	n/a	1620	–62
Chimney site	2	280	1620	–57
	3	280	1620	–121
	4	250	1620	–122
Site A	1	n/a		
	2	60	1655	–178
	3	130	1635	–45
	4	100	1625	–115
a minor site	1	n/a		
	2	n/a		
	3	–90	1620	–138
	4	–110	1620	–142

* Polarisation is 0 at the 3 o'clock direction; it increases in the anti-clockwise direction. For example, polarisation is –90 degrees for a downward dipole.

Supplementary document 1

Appendix to the *Materials and Methods* section.

Instruments. Our tool, developed to conduct self-potential surveys in marine environments[1] (Fig. 2), consists of an electrode array and a data acquisition unit installed on a deep-tow apparatus. A fibre-reinforced plastic (FRP) rod (30 m long, 1 cm diameter in this study) bends and recovers elastically to keep the rod straight. Thereby the sensor distance is maintained. Five non-polarised Ag-AgCl electrodes[2] are mounted at equal intervals of 5 m along the rod (Fig. 2). A common electrode is placed between the second and third electrodes. The use of numerous electrodes is important for observing marine environments because of redundant data acquisition, but only two electrodes can measure the self-potential. We placed a sea sinker at the tail of the rod to stabilise it. During surveys, the electrostatic potential of each electrode was recorded using a stand-alone precise (24 bit) voltage meter. The recorder contains a battery and requires no external power supply. We used 1 Hz resampled data extracted from the original 50 Hz data.

Positioning. The deep-tow position (in three dimensions; Fig. 2) was monitored using a super-short baseline (SSBL) ranging between the deep-tow and the vessel. The time interval of ranging is 8 s. The accuracy of the SSBL ranging is within 1% of the slant range. It is ±20 m in the present case with the deep-tow. The deep-tow depth is also monitored using a pre-calibrated depth meter, with a time interval of 1 s. For analysis, the deep-tow depth obtained from the depth meter is used because it is more stable and

accurate (< 1 m in specifications) than that obtained from SSBL ranging. To calibrate the depth meter, a one-dimensional profile of sound speed is obtained using a CTD launcher system. The deep-tow altitude is monitored using an acoustic altimeter, with a time interval of 1 s. The sum of the measured depth and altitude is the seafloor depth below the deep-tow apparatus.

The electrode rod tail position is also monitored by SSBL ranging. The relative position of the rod can be estimated using the two SSBL positions by assuming the rod as straight. To obtain a smooth time series of the positions of the deep-tow and the tail of the rod, least-squares spline approximation is applied to the raw data.

Electric field. The electric field in the direction of the rod is calculable using any combination of two electrodes (out of five electrodes) divided by the distance between them, with the position of measurements being defined at its centre. In general, using a long sensor distance gains sensitivity, i.e. the signals become larger, but the positioning becomes inaccurate, whereas taking a short sensor distance improves the positioning accuracy, but the sensitivity worsens. We chose the shortest sensor distance of 5 m because we found that the electric field is not constant along the 30-m-long rod, particularly when the deep-tow altitude is low (e.g. 5 m). This difficulty related to the electric field probably occurs because the electrode rod is not straight. Therefore, it reflects rapid changes in the deep-tow altitude. The electrode cable responds to local self-potential signals near sulphide mounds with spatial scale of approx. 30 m. Results show that these four combinations of 5-m-spacing electrode pairs produce the same electric field if the location of measurements is given properly and if an offset value

(assumed to be constant) inherent to each electrode pair is subtracted (*Supplementary* Fig. S1). Therefore, we can assume that the self-potential signal is homogeneous with the spatial scale of approx. 5 m.

Cable angle. The slope angle of the rod is calculated using the SSBL positions of the deep-tow and the tail of the rod with the rod assumed as straight. Although the SSBL ranging has an absolute accuracy of only about 1% of the slant range (20 m in the present case), the relative depth between the two transponders is expected to be exceedingly accurate. Even with a sea sinker, the rod tail was typically 10 m shallower than the deep-tow when towed at a constant water depth. The angle is arcsin(10/30) or approx. 19.5°. This angle value does not affect the result significantly because cos(19.5°) is approx. 0.94. This angle is increased or decreased when the deep-tow is descending or ascending. The cable angle relative to the deep-tow heading tends to become small (*Supplementary* Figs. S2 and S3), although it has a delay reflecting the elastic behaviour of the electrode cable. Given the relative position of the sensors on the rod and the slope angles of the deep-two survey line and the cable, the position of each electrode can be estimated in the three-dimensional space. The effects of ignoring information of the cable position are investigated in *Supplementary document 3*.

Effective self-potential. As a visual reference, the calculated electric field is integrated along the dive tracks to reveal the self-potential along the dive tracks. The measured electric field is not parallel to the dive track in general. Therefore, we approximate the

electric field along the dive track as equal to the observed electric field. The "effective" self-potential is therefore calculated as

$$V = V_{\rm ref} + \int_{x_{\rm ref}}^{x} E_{\rm obs} \frac{dx}{\cos\theta}, \tag{S1}$$

where θ represents the slope angle of the survey line (i.e. $\cos\theta = 1/\sqrt{1+(dz/dx)^2}$), and subscript 'ref' denotes a reference point that is distant from ore bodies. This integration works well for practical uses because both the initial and final self-potentials are found to be approx. 0 mV (upper panels, Figs. 3 and 4). As described in the *Results* section, we do not use this "effective" self-potential to detect the location of sources for the observed self-potential signals, but instead use the observed electric field in the direction of the rod ($E_{\rm obs}$; *Supplementary* Fig. S1) for analysis of the probability tomography method. In the present survey, the calculated effective self-potential is zero both before and after passing sulphide mounds, indicating that the data obtained with an error in the rod angle are sufficiently accurate for our purposes.

Method of detecting self-potential signals. The probability tomography method[3–6] is a kind of cross-correlation method to produce images of the source locations of the self-potential signals in a probabilistic manner. The method was first limited to an electric current monopole source below a flat surface[3]. Then the effects of topography[4] and dipole sources[5] were included. Assuming an electric current dipole as a first approximation to represent an ore body[5,7], we obtain the expression for cases in which the direction of the measured electric field is not parallel to the survey line[6] (Fig. 2 shows the geometric configuration.).

The occurrence probability of a unit-strength electrical current dipole (η_i; $i = V$ for a vertical dipole and $i = H$ for a horizontal dipole) located at (x_q, z_q) is calculated by taking cross-correlation between the observed electrical field in the direction of the rod (E_{obs}) and the synthetic electric field induced by a unit-strength dipole ($\Im_i$; $i = V, H$). The result is

$$\eta_i(x_q, z_q) = \frac{\int E_{obs}(x)\, \Im_i(x - x_q, z(x) - z_q) \frac{dx}{\cos\theta}}{\sqrt{\int \{E_{obs}(x)\}^2 \frac{dx}{\cos\theta}} \sqrt{\int \{\Im_i(x - x_q, z(x) - z_q)\}^2 \frac{dx}{\cos\theta}}}, \tag{S2}$$

where $\theta(x, z(x))$ is the slope angle of the dive track, $\varphi(x, z(x))$ is the slope angle of the electrode array, and

$$\Im_i = -\left(\cos\varphi \frac{\partial S_i}{\partial x} + \sin\varphi \frac{\partial S_i}{\partial z}\right), \tag{S3}$$

with S_i ($i = V, H$) is the self-potential field induced by an electric current dipole. Here we assume for simplicity that the survey line is along the x direction (north–south). The use of E_{obs} instead of V_{eff} produces accurate results because we can equate the direction of the electric field induced by the synthetic electric current dipole ($\Im_i$) to the direction of E_{obs}.

If the effect of electrical conductivity contrast is ignored, then S_i has the following form[5,6]:

$$(S_V, S_H) = \frac{(x - x_q,\, z(x) - z_q)}{\left\{(x - x_q)^2 + (z(x) - z_q)^2\right\}^{3/2}}. \tag{S4}$$

This simplification gives no effect for the case of a flat seafloor with a layered electrical conductivity structure. Considering the seafloor topography, this simplification might also be justified at a first order, even though the electrical conductivity in shallow sediment might vary by approximately five times within the uppermost few tens of

metres. Synthetic tests show that contrasts in electrical conductivity of the one-order of magnitude can be ignored in the present analysis when the required accuracy is approx. 10 m (*Supplementary document 2*). For more precise estimations, S_i should be calculated numerically or an inversion should be performed[8].

Scanning the location of an electric current dipole (x_q, z_q) calculates the occurrence probability of a dipole. In the present study, the spacing is 10 m in the horizontal direction and 5 m in the vertical direction. The value of η_i ($i = V, H$) is the occurrence probability of an electric current dipole in the vertical or horizontal direction. In Figs. 6 and S4, the value of

$$\eta_T \equiv \sqrt{(\eta_V)^2 + (\eta_H)^2} \qquad \text{(S5)}$$

is the overall probability of an electric current dipole. The polarisation direction is defined as the following unit vector:

$$\left(\frac{\eta_H}{\sqrt{(\eta_H)^2 + (\eta_V)^2}}, \frac{\eta_V}{\sqrt{(\eta_H)^2 + (\eta_V)^2}} \right). \qquad \text{(S6)}$$

References

1. Goto, T. *et al.* Electromagnetic survey around the seafloor massive sulfide using autonomous underwater vehicle. *Proc. 11th SEGJ Intl. Symposium* 342–345, doi:10.1190/segj112013-087 (SEGJ, 2013).
2. Filloux, J. H. Instrumentation and experimental methods for oceanic studies in *Geomagnetism Vol. 1* (ed. Jacobs, J. A.) 143–248 (Academic Press, 1987).
3. Patella, D. Introduction to ground surface self-potential tomography. *Geophys. Prospect.* **45**, 653–681 (1997).

4. Patella, D. Self-potential global tomography including topographic effects. *Geophys. Prospect.* **45**, 843–863 (1997).

5. Revil, A., Ehouarne, L. & Thyreault, E. Tomography of self-potential anomalies of electrochemical nature. *Geophys. Res. Lett.* **28**, 4363–4366 (2001).

6. Revil, A., Naudet, V., Nouzaret, J. & Pessel, M. Principles of electrography applied to self-potential electrokinetic sources and hydrogeological applications. *Water Resource Res.* **39**, 1114, doi:10.1029/2001WR000916 (2003).

7. Sato, M. & Mooney, H. M. The electrochemical mechanism of sulfide self-potentials. *Geophysics* **25**, 226–249 (1960).

8. Heinson, G., White, A., Robinson, D. & Fathianpour, N. Marine self-potential gradient exploration of the continental margin. *Geophysics* **70,** G109–G118 (2005).

Supplementary document 2

Effects of electrical conductivity contrast on estimating the electric dipole source depth.

Herein, we demonstrate that the contrast of electric conductivity can be ignored in analyses when the source location is emphasized.

Numerical Model. We calculate the electric potential field induced by a dipolar electric current source below the seafloor in the presence of seafloor topography and electrical conductivity contrast. We resolve this difficulty using three-dimensional finite element method. An electric dipole is represented as a spatial derivative of the delta function[9]. The equation to be solved is given as

$$\vec{\nabla}\cdot\left(\sigma\vec{\nabla}\phi\right)=I_0\left.\frac{\partial\delta}{\partial n}\right|_{\vec{r}=\vec{r}_0}, \quad \text{(S7)}$$

where ϕ stands for the electric potential (self-potential in our case), σ signifies electrical conductivity, I_0 denotes the source intensity, δ denotes the delta function, r_0 represents the location of an electric dipole, and n is the direction of polarisation. For simplicity, the Dirichlet boundary condition with zero potential is applied for all outer boundaries. An electric dipole is represented as a spatial derivative of a *discrete* delta function[9].

The calculation area is a cube of 1000 m with the upper half and lower half respectively including the ocean and sediment (Fig. S7a). This area is discretized by 100 unevenly sized (approx. 1.25 m from the seafloor) tri-linear hexahedron elements in each dimension. We restrict ourselves to performing calculations corresponding to the largest

mound encountered in the present study (the south site). The seafloor topography (Fig. S7b) is approximated as shown below.

$$z_{\mathrm{topo}} = \Delta z \exp\left\{ -\left(\frac{x - x_0}{\alpha_x} \right)^2 - \left(\frac{y - y_0}{\alpha_y} \right)^2 \right\} + z_{\mathrm{ref}} \tag{S8}$$

Therein, $\Delta z = 30$ m, $\alpha_{\xi} = \alpha_y = 50$ m, and $z_{\mathrm{ref}} = 0$.

We assume electrical conductivity of three types with seawater conductivity of 3 S/m: (1) constant electrical conductivity; (2) electrical conductivity of the sediment is 0.1 times that of seawater, which is probably an extreme case for shallow sediments; (3) electrical conductivity of the sediment is half that of seawater at the seafloor, decreasing exponentially with the e-folding depth of 50 m. A downward-polarised dipolar source is placed at 10, 30, or 50 m beneath the topographic high.

We estimate the source location using probabilistic tomography described in the main text. The spacing is 1 m in both the horizontal and vertical directions. We monitor the electric potential 5 m above the seafloor and at 50 m high relative to the topographic low (dashed curves in Fig. S7).

Numerical results. With constant electrical conductivity, the resultant electric field (Fig. S8a; a 50-m-deep source is imaged using a 5-m-height survey line) is fundamentally the same as the analytical solution in an infinite space[10], as

$$\phi \propto \frac{z - z_0}{\left\{ (x - x_0)^2 + (y - y_0)^2 + (z - z_0)^2 \right\}^{3/2}} \quad . \tag{S9}$$

This equation shows the effect of the Dirichlet boundary condition because ϕ is inversely proportional to the square of the distance from the source. We confirmed that the

probabilistic tomography can estimate the source depth exactly (Table S3).

With piecewise-constant electrical conductivity in which electrical conductivity of the sediment is 0.1 times that of seawater, the resultant electric field is increased significantly (with the same source intensity; see equation (S7)). In this case, probabilistic tomography that ignores electric conductivity contrast produces almost true source location (Table S3). Similar results are obtainable for cases with a more realistic electric conductivity profile (Fig. S8b and Table S3). In these examples, the estimation error is within 10% or 5 m for most cases. Therefore, information related to electric conductivity is unimportant to estimate the source location, but the electric conductivity is of particular importance when the source intensity is considered.

References

9. Schimpf, P. H., Ramon, C. & Haueisen, J. Dipole Models for the EEG and MEG. *IEEE Trans. Biomed. Eng.* **49**, 409–418 (2002).

10. Griffiths, D. J. *Introduction to Electrodynamics, Fourth edition.* 608 pp. (Pearson, 2013).

Table S3. Estimated source depth of an embedded electric dipole

$\sigma_{\text{sediment}}/\sigma_{\text{seawater}}$	Prescribed source depth (m)	Estimated depth by a 5-m-height survey line* (m)	Estimated depth by a 50-m-height survey line* (m)
	10	11	10
1	30	30	30
	50	50	50
	10	10	9
0.1	30	30	29
	50	51	51
$0.5\exp\left(-\frac{z_{\text{sf}}(x,y)-z}{50\ \text{(m)}}\right)$	10	10	9
	30	29	27
	50	47	44

* Calculations were done with 1-m intervals in the vertical and horizontal directions.

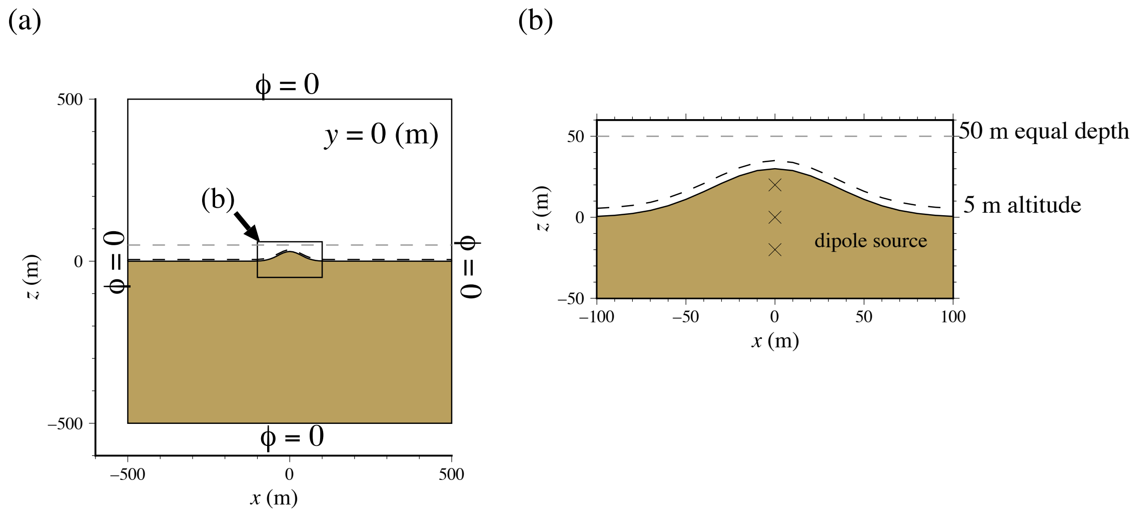

Figure S7. Geometry for finite element modelling: (a) Calculation domain of a 1000 m cube with the small black square denoting the area shown in (b). The lower brown area represents the sediment. The upper white area represents seawater. Dashed curves denote survey lines. (b) Magnified calculation domain. Crosses (10, 30, and 50 m below the topographic high) correspond to locations of a prescribed electric dipole.

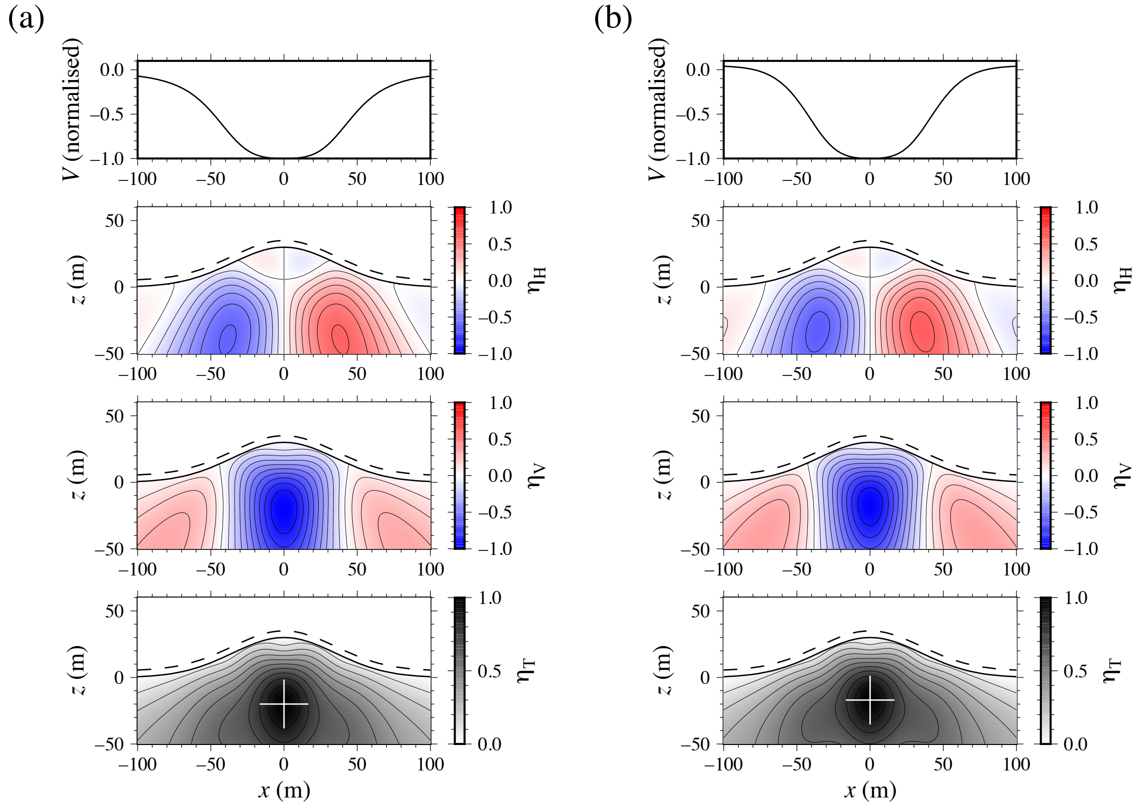

Figure S8. Results of finite element modelling: Typical results with (a) a constant electrical conductivity and (b) depth-dependent electrical conductivity. Both cases assume a downward electric dipole 50 m below the topographic high. The survey height is 5 m above the seafloor. (Upper panel) Resultant self-potential. (Second and third panel) Occurrence probability of the horizontal and vertical dipoles (Colour meanings are shown in the legend at right). (Lower panel). (b) Overall occurrence probability. The white plus sign is the estimated source location.

Supplementary document 3

Effects of errors on estimating the electric dipole source depth.

Here we consider how errors involved in the observation affect the estimated source locations of the observed self-potential anomalies. We present two selected examples corresponding to Track 1 of the western survey line (altitude of approx. 50 m) and Track 4 of the eastern survey line (altitude of approx. 5 m). See also Table S1 for the typical noise levels of the respective observed values.

Electrode noise. Electrode noise is extremely low (< 0.1 mV). Normally it presents no difficulties. Usage of numerous electrodes is necessary because the repair of electrodes is not an easy task once they are deployed in the ocean. Adding Gaussian random noise with a variance of 1 mV decreases the occurrence probability. However, the locations at which dipoles are expected can be imaged. Non-zero noise might affect predictability.

Deep-tow positioning. Horizontal positioning is ±20 m as the worst estimate, which is insufficiently accurate in the absolute sense, but sufficient in the relative sense. For this study, the dive track is smoothed in advance so that integration (equation (S1) in *Supplementary document 1*) can be done. Horizontal shifts in the position should be calibrated using the bathymetry of the target area. A good guide is a correlation between topographic highs and locations in which the self-potential signals are distinct.

Cable angle. The cable angle (φ) is an inaccurate factor involved in the observation. Although the calculated angle is calculated as stable because it is calculated using the relative depth between two transponders (middle panels in Figs. S2 and S3), the cable is not guaranteed to be straight. As an example for the worst case, we set the cable angle to 0. Then we performed the same analysis corresponding to Figs. 6 and S4. The result is almost identical to the original case with the obtained cable angle.

Slope angle of the deep-tow survey line. The slope angle of the survey line (θ), which is calculated from the time derivative of the transponder and depth-meter records, shows scattering (upper panels in Figs. S2 and S3). High-altitude surveys give this angle as zero. As an example for the worst case, we set this angle to 0. Furthermore, the cable angle (φ) was set to zero. Both cases produce almost identical results to those obtained in the original case (cf. Fig. S9a and Fig. 6a for Track 2 of the western survey line, and Fig. S9b and Fig. 6d for Track 4 of the eastern survey line).

Deep-tow depth. However, when the deep-tow depth is set as constant (1600 m), the result becomes worse when the deep-tow altitude changes with time (cf. Fig. S9c and Fig. 6c for Track 2 of the western survey line, and Fig. S9d and Fig. 6d for Track 4 of the eastern survey line). The distance between the source and the observed points is important for analysis because a dipole has strong dependence on the distance (inverse-square relation). The deep-tow depth is obtained very accurately (approx. 1 m). The test described above is unrealistic. However, if the depth meter is broken, then imaging cannot be performed very well.

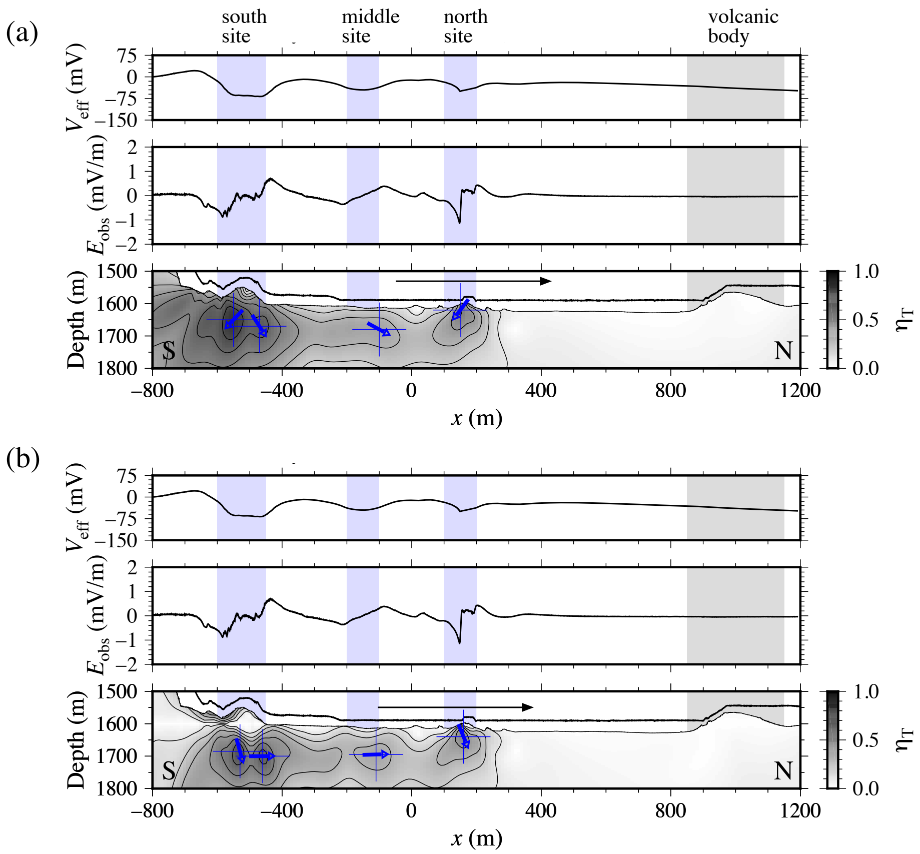

Figure S9. Results of sensitivity analysis for exploring the source of the observed self-potential anomalies: (a) and (b) depict Track 2 of the western survey line. (c) and (d) show Track 4 of the eastern survey line. (a) and (c) ignore the slope angles of the survey line and the electrode cable, whereas (b) and (d) take a constant water depth (1600 m). (Upper panel) Effective self-potential calculated from the electric field. (Middle panel) Observed electric field. (Lower panel) Overall occurrence probability of an electric current dipole (colour scales are shown at the right of the respective panels). The contour interval is 0.1. Black curves denote the deep-tow dive track. The blue arrow corresponds to the polarisation direction of dipoles, where the occurrence probability takes the local maximum.

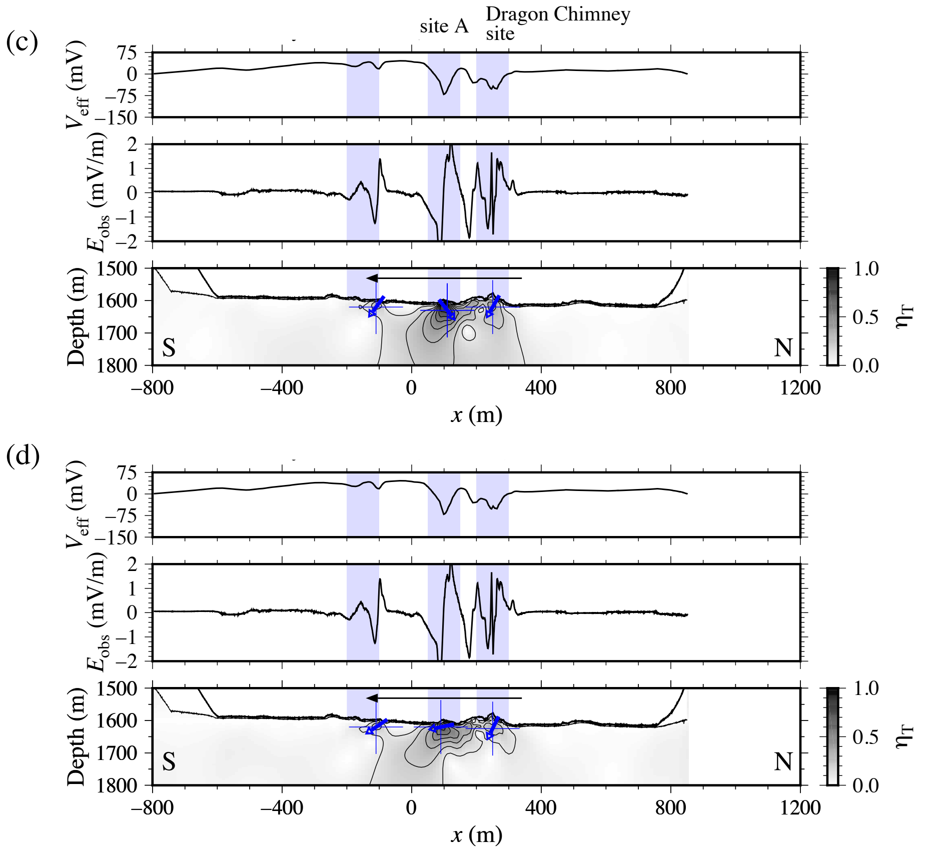

Figure S9. (Continued)
